# IL-27-induced PD-L1^high^Sca-1^+^ innate lymphoid cells suppress contact hypersensitivity in an IL-10-dependent manner

**DOI:** 10.1038/s12276-024-01187-1

**Published:** 2024-03-01

**Authors:** Keun Young Min, Do-Kyun Kim, Min Geun Jo, min Yeong Choi, Dajeong Lee, Jeong Won Park, Young-Jun Park, Yeonseok Chung, Young Mi Kim, Yeong-Min Park, Hyuk Soon Kim, Wahn Soo Choi

**Affiliations:** 1 Department of Immunology, School of Medicine, Konkuk University, Chungju, 27478 Republic of Korea; 2 Korea Zoonosis Research Institute, Jeonbuk National University, Iksan, 54531 Republic of Korea; 3 Department of Biomedical Sciences, College of Natural Science and Department of Health Sciences, The Graduate School of Dong-A University, Busan, 49315 Republic of Korea; 4 College of Pharmacy, Jeju National University, Jeju, 63243 Republic of Korea; 5 Research Institute of Pharmaceutical Sciences, College of Pharmacy, Seoul National University, Seoul, 08826 Republic of Korea; 6 Department of Preventive Pharmacy, College of Pharmacy, Duksung Women’s University, Seoul, 01369 Republic of Korea

**Keywords:** Innate lymphoid cells, Allergy

## Abstract

Innate lymphoid cells (ILCs) play an important role in maintaining tissue homeostasis and various inflammatory responses. ILCs are typically classified into three subsets, as is the case for T-cells. Recent studies have reported that IL-10-producing type 2 ILCs (ILC2_10_s) have an immunoregulatory function dependent on IL-10. However, the surface markers of ILC2_10_s and the role of ILC2_10_s in contact hypersensitivity (CHS) are largely unknown. Our study revealed that splenic ILC2_10_s are extensively included in PD-L1^high^Sca-1^+^ ILCs and that IL-27 amplifies the development of PD-L1^high^Sca-1^+^ ILCs and ILC2_10_s. Adoptive transfer of PD-L1^high^Sca-1^+^ ILCs suppressed oxazolone-induced CHS in an IL-10-dependent manner Taken together, our results demonstrate that ILC2_10_s are critical for the control of CHS and suggest that ILC2_10_s can be used as target cells for the treatment of CHS.

## ILC210: a potential new target for contact hypersensitivity treatment

Innate lymphoid cells (ILCs - a type of immune cell) are important for tissue health and immunity. However, the function of a specific group of these cells, known as IL-10+ ILCs, in skin inflammation is unclear. Scientists have found that a particular type of IL-10+ ILCs, named PD-L1highSca-1+ ILCs, can reduce skin inflammation in a mouse model of contact hypersensitivity (CHS - a type of allergic reaction). The research showed that these cells increase during inflammation and can lessen the activity of other immune cells. The scientists also discovered that a protein named IL-27 is vital for the creation of these cells. Hence, this research offers new understanding into the role of IL-10+ ILCs in skin inflammation and could potentially lead to new skin disease treatments.

This summary was initially drafted using artificial intelligence, then revised and fact-checked by the author.

## Introduction

Recent studies have shown that innate lymphoid cells (ILCs) play a critical role in maintaining tissue homeostasis and protective immunity^1^. ILCs develop in the bone marrow, as is the case for other common lymphoid progenitor (CLP)-originated immune cells, such as T cells, after which they migrate to peripheral tissues to be extensively distributed on the mucosal surface and in lymphoid tissues^2^. Generally, individual ILC subsets express signature cytokines and transcription factors, indicating that they function similarly to T-cell subsets. For example, three typical types of ILCs—ILC1, ILC2, and ILC3—mirror Type 1 T helper (Th1), Th2, and Th17 cells^3,4^. ILC1s, which are characterized by the expression of the T-box transcription factor (T-bet), secrete interferon-γ (IFN-γ) and play a role in type 1 immune responses^5,6^. ILC2s, which are characterized by the expression of transcription factors such as GATA-binding protein 3 (GATA3) or RAR-related orphan receptor alpha (RORα), secrete Th2 cytokines, including IL-5 and IL-13. ILC2s predominantly play a role in type 2 immune responses triggered by parasites or allergens. ILC3s, which are characterized by the expression of RORγt, are responsible for secreting cytokines such as IL-17 and IL-22. Therefore, ILC3s play a crucial role in the immune response, specifically in relation to eliminating extracellular microbes^7–12^.

The skin, the body’s largest barrier organ, provides protection against trauma, thermal changes, and external factors. The skin is composed of the epidermis, dermis, and subcutaneous tissue, each of which has distinct functions and cell distributions. Like in other tissues, the skin has a varied distribution of ILCs across different regions. In the epidermis, ILC3s predominate, while the dermis harbors a combination of ILC2s and ILC3s, with ILC2s primarily residing in subcutaneous tissue^13–15^. Previous studies have suggested that various subsets of ILCs may play crucial roles in maintaining skin homeostasis. For instance, the number of ILC2s is higher in the skin lesions of atopic dermatitis (AD) patients compared to healthy control skin tissues, and ILC2s are considered a pivotal factor that promotes disease in the MC903-induced atopic dermatitis mouse model^16^. Conversely, other research has indicated that ILC2s may act as negative regulators within the DNCB-induced contact hypersensitivity (CHS) mouse model^17^. In addition, the NKp44^+^ ILC3 population has been observed to be more abundant in the lesional skin and peripheral blood of psoriasis patients than in that of healthy individuals^18^.

Moreover, emerging evidence has demonstrated that a distinct subset of ILCs secrete IL-10. We first reported the expression of IL-10 in splenic ILCs of lineage-negative (Lin^−^)CD45^+^CD127^+^, and demonstrated that significant changes in the IL-10^+^ ILC population occurred in mice with oxazolone (OXZ)-induced CHS^19^. Subsequently, Wang et al. extensively revealed the presence of an intestinal IL-10-producing ILC subset termed regulatory ILCs (ILCregs). They argued that ILCregs develop through an inhibitor of DNA binding (Id)3-dependent mechanism and inhibit the activation of ILC1s and ILC3s to suppress intestinal inflammation, and the generation of ILCregs is activated by transforming growth factor (TGF)-β1^20^. Another study reported that the IL-2-mediated mechanism promotes IL-10 production by lung-derived ILC2 subsets, also known as ILC2_10_s. They further demonstrated that IL-10 secreted from ILC2_10_s suppressed eosinophil recruitment into the lung^21^. Furthermore, Morita et al. introduced the IL-10-producing ILC2 subset in humans, noting that retinoic acid (RA) stimulates the production of ILC2_10_s. In addition to suggesting a new molecule, RA, for the activation of ILC2_10_s, the study showed that ILC2_10_s can suppress not only other ILC subsets but also the activation of T cells in an IL-10-dependent manner^22^. Another report suggested that cultured intestinal ILC2s had significantly increase IL-10 production via IL-2, but intestinal IL-10^+^ ILCregs were rarely detected in intestinal inflammation^23^. Taken together, despite the apparent existence of a regulatory subset of ILCs capable of secreting IL-10, these results show that the phenotypic features of IL-10-producing ILCs remain largely unknown. Furthermore, clarifying the role of these less-studied IL-10^+^ ILCs in skin inflammation, compared to other ILC subsets, is important.

In this study, we observed that a distinctive PD-L1^high^Sca-1^+^ ILC subset comprises ILCs that are primarily involved in the production of IL-10 in a CHS mouse model. We further demonstrated that IL-27 signaling is critical for the formation of IL-10^+^ PD-L1^high^Sca-1^+^ ILCs in vitro and in vivo and that the adoptive transfer of PD-L1^high^Sca-1^+^ ILCs suppresses CHS in an IL-10-dependent manner.

## Materials and methods

### Mice

WT C57BL/6 (B6) mice were purchased from Orient Bio, Inc. (Gyeonggi-do, Korea). B6.129P2-Il10^tm1Cgn^/J (*Il10*^*−/−*^, Jax Stock #002251), B6.129S6-Il10^tm1Flv^/J (*IL-10-GFP*; *tiger*, Jax Stock #008379), and B6N.129P2-Il27ra^tm1Mak^/J (*Il27ra*^*−/−*^, Jax Stock #018078) of the C57BL/6 background were purchased from The Jackson Laboratory (Bar Harbor, ME, USA). Male mice aged 6 weeks were used for all experiments. All mice were bred and maintained in a pathogen-free facility at Konkuk University (Seoul, Korea). All animal experiments were approved by the Institutional Animal Care and Use Committee (IACUC) of Konkuk University.

### Induction of CHS

CHS was induced with a previously described method^24^. In brief, mice were sensitized on the shaved hind flank for two consecutive days with 25 μl of oxazolone (OXZ; 100 mg/ml; Sigma‒Aldrich) in acetone (ACE)/olive oil (4:1, v/v). On day 5, mice were challenged by the application of 10 μl of OXZ (10 mg/ml) in acetone/olive oil (4:1, v/v) to the ear. At the completion of OXZ challenge, the thickness of the ear was measured every day for 4 days by three blinded independent observers. On day 7, the cells from the isolated lymphoid and ear tissues were analyzed via flow cytometry and real-time PCR.

### Flow cytometry and cell sorting

Single cells isolated from the spleen, cervical lymph node (cLN) and ear were stained with a Zombie NIR™ Fixable Viability Kit (BioLegend) to exclude dead cells. Before staining for cell surface markers, the cells were incubated with an anti-CD16/32 antibody for 5 min to block surface Fc receptors. The following antibodies against surface proteins were utilized: antibodies against B220 (RA3-6B2), c-Kit (2B8), CCR9 (eBioCW-1.2), CD1d (1B1), CD2 (RM2-5), CD4 (GK1.5), CD5 (53-7.3), CD8a (53-6.7), CD11b (M1/70), CD11c (N418), CD19 (eBio1D3), CD21/CD35 (eBio8D9), CD23 (B3B4), CD25 (PC61.5), CD49b (DX5), CD62L (MEL-14), CD103 (2E7), CD127 (A7R34), CTLA-4 (HMCD15201), FceRI alpha (MAR-1), F4/80 (BM8), Gr-1 (RB6-8C5), ICOS (C398.4 A), LAP (TW7-16B4), LAG-3 (eBioC9B7W), Ly-6C (HK1.4), NKp46 (29A1.4), Sca-1 (D7), ST2 (RMST2-2), TCR beta (H57-597) and TER-119 (TER-119) were obtained from Invitrogen (Carlsbad, CA); antibodies for CD3 (17A2), CD18 (M18/2), CD45 (30-F11), CD206 (C068C2), CD210 (IL-10 receptor)(1B1.3a), LAP (TW7-16B4) and PD-L1 (10 F.9G2) were purchased from BioLegend (San Diego, CA); antibodies against 2B4 (2B4), CD27 (LG.3A10), CD72 (K10.6), and NK1.1 (PK136) were from BD Biosciences (San Jose, CA); and antibody specific to IL-27R alpha (263503) was obtained from R&D Systems (Minneapolis, MN). For intracellular staining, the following antibodies were used: antibodies against IL-10 (JES5-16E3), IFN-γ (XMG1.2), IL-17A (eBio17B7), T-bet (eBio4B10), GATA-3 (TWAJ), RORγt (AFKJS-9) and Foxp3 (FJK-16s) were obtained from Invitrogen; an antibody against IL-17A (TC11-18H10) was obtained from BD Biosciences; and an anti-IL-27p28 (MM27-7B1) antibody was obtained from BioLegend. The cells were then stained with surface markers.

For intracellular cytokine staining, cells were stimulated with PMA (50 ng/ml; Sigma‒Aldrich, St. Louis, MO), ionomycin (500 ng/ml; Sigma‒Aldrich), and brefeldin A (3 µg/ml; eBioscience) (for IFN-γ and IL-17A); monensin (2 μM; eBioscience) (for IL-10); and lipopolysaccharide (10 μg/ml; Sigma‒Aldrich) and monensin (2 μM; eBioscience) (for IL-27p28) for 4 h. Then, the cells were fixed and permeabilized using fixation buffer and permeabilizing solution 2 according to the manufacturer’s instructions (BD Biosciences, San Jose, CA). For transcription factor analysis, cells were subjected to intranuclear staining using the foxp3/transcription factor staining buffer set (eBioscience) according to the manufacturer’s instructions. A FACSCanto II (BD Biosciences) instrument was used for flow cytometry analysis. The data were analyzed with FlowJo software version 10 (TreeStar). To isolate GFP^+^ ILCs (Lin^−^CD45^+^CD127^+^GFP^+^ from *tiger* mice), GFP^−^ ILCs (Lin^−^CD45^+^CD127^+^GFP^−^ from *tiger* mice), PD-L1^high^Sca-1^+^ ILCs (Lin^−^CD45^+^CD127^+^PD-L1^high^Sca-1^+^ from WT or *Il10*^*−/−*^ C57BL/6 mice), or PD-L1^low/−^Sca-1^−^ ILCs (Lin^−^CD45^+^CD127^+^PD-L1^low/−^Sca-1^−^ from WT C57BL/6 mice), a single-cell suspension of splenocytes was stained with the indicated antibodies and sorted via FACSAria (BD Biosciences) with a sorting purity of greater than 95%.

### Single-cell preparation

The spleen and cLN were prepared using 70 μm strainers (SPL Life Sciences, Gyeonggi-do, Korea). Ear tissues were cut into small pieces and digested at 37 °C for 40 min in RPMI (Roswell Park Memorial Institute)-1640 medium containing 100 μl of enzyme D, 50 μl of enzyme R, and 12.5 μl of enzyme A from a tumor dissociation kit (Miltenyi Biotec, Bergisch Gladbach, Germany). After digestion, 2 ml of RPMI-1640 medium was added, and dissociation commenced by the gentleMACS program_B_01. Following dissociation, the tissues were passed through 70 μm strainers. After centrifugation, the resulting cell pellet was washed with sterile phosphate buffered saline (PBS), centrifuged, and resuspended in RPMI-1640 medium.

### Adoptive transfer of ILC subsets

IL-10-GPF^+^ or IL-10-GPF^–^ ILCs were isolated from *tiger* mice. PD-L1^high^Sca-1^+^ or PD-L1^low/−^Sca-1^−^ ILCs were isolated from WT or *Il10*^*−/*−^ C57BL/6 mice. Each ILC subset (5 ×10^4^ cells per recipient mouse, i.v., one day before sensitization; 5 × 10^3^ cells per ear of recipient mouse, i.d., one day before sensitization) was injected into WT C57BL/6 or *Il10*^*−/−*^ mice.

### T-cell subsets cocultured with ILCs

Splenic CD4^+^ and CD8^+^ T cells from WT C57BL/6 mice were enriched via a CD4 or CD8a T-cell isolation kit (Miltenyi Biotec) according to the manufacturer’s instructions. Enriched CD4^+^ and CD8^+^ T cells (5 × 10^4^ cells/well) were cultured in round-bottom 96-well plates with PD-L1^high^Sca-1^+^ ILC subsets (2.5 × 10^4^ cells/well) derived from WT or *Il10*^*−/−*^ C57BL/6 mice for 24 h in the presence of anti-CD3ε (2 μg/ml, BioLegend) and anti-CD28 (2 μg/ml, eBioscience) antibodies. The expression of IFN-γ and IL-17A on CD4^+^ and CD8^+^ T cells was examined by flow cytometry.

### In vitro culture of ILCs with cytokines

The negative selection of spleen cells from WT B6 or *Il10*^*−/−*^ C57BL/6 mice was performed using a lineage cell depletion kit (Miltenyi Biotec) according to the manufacturer’s instructions. Sort-purified Lin^−^ cells (1 × 10^5^ cells per well) were cultured in complete media in 96-well plates for 12 h in the presence of the indicated cytokines.

### Measurement of cytokine secretion

Lin^−^ cells (1 × 10^5^ cells per well) sorted by using a lineage cell depletion kit (Miltenyi Biotec) were incubated in 96-well plates for 12 h in the presence of the indicated cytokines or IL-27 (1, 10 and 100 ng/ml; Biolegend). IL-10 levels were quantified using an IL-10 mouse uncoated ELISA kit (Thermo Fisher, Waltham, MA) according to the manufacturer’s instructions.

### In vivo recombinant IL-27 or IL-27 mAb treatment

For the rmIL-27p28 injection study, rmIL-27 (200 ng per mouse; BioLegend) or sterile PBS was injected intravenously (i.v.) or intraperitoneally (i.p.) into WT B6 mice every day for 5 days before challenge. For IL-27p28 neutralization in vivo, an anti-IL-27p28 mAb (250 μg per mouse, BioXCell) or a mouse IgG2a isotype control antibody (250 μg per mouse, BioXCell) was injected into the peritoneal cavity of WT B6 mice on days 4 and 5 after the first sensitization.

### Real-time PCR

Snap-frozen mouse ear tissues were ground to powder, and PD-L1^high^Sca-1^+^ ILCs and PD-L1^low/−^Sca-1^−^ ILCs were sorted by FACSAria. RNA was extracted using an easyTM-spin Total RNA Extraction Kit (iNtRON, Burlington, MA) according to the manufacturer’s instructions. cDNA synthesis was carried out with a Tetra cDNA Synthesis Kit (Bioline, London, UK). Real-time PCR was performed using LightCycler 480 SYBR Green I Master Mix (Roche, Basel Switzerland). PCR was performed using the following primers: mouse *Tbx21* forward 5′-CTGGAGCCCACAAGCCATTA-3′ and reverse 5′-TTTCCACACTGCACCCACT-3′; mouse *Gata3* forward 5′-CGCTACGGTGCAGAGGTATC-3′ and reverse 5′-GAGGGTAAACGGACAGAGGC-3′; mouse *Rorc* forward 5′-GCAGGGCCTACAATGCCAAC-3′ and reverse 5′-GAACCAGGGCCGTGTAGAGG-3′; mouse *Il1b* forward 5′-TGGAGAAGCTGTGGCAGCTA-3′ and reverse 5′-GAACGTCACACACCAGCAGGT-3′; mouse *Ifng* forward 5′-GCCACGGCACAGTCATTGA-3′ and reverse 5′-TGCTGATGGCCTGATTGTCT-3′; and mouse *actinb* forward 5′-AAGTGTGACGTTGACATCCG-3′ and reverse 5′-GATCCACATCTGCTGGAAGG-3′. Gene expression was normalized to the expression of the housekeeping gene *actinb* by an n-fold difference.

### Histology and immunofluorescence

For histopathological analysis, ear tissues from mice were fixed with 4% paraformaldehyde (PFA) in PBS, dehydrated and embedded in paraffin. The 5-ɥm tissue sections were stained with hematoxylin and eosin (H&E). To determine the importance of the adoptive transfer of GFP^+^ ILCs, spleen, cLN, and ear tissue sections were blocked with 10% normal horse serum for 1 h and stained with the GFP polyclonal antibody Alexa Fluor 488 (Thermo Fisher). 4′,6′-Diamidino-2-phenylindole (DAPI) (Thermo Fisher) was used as a counterstain to label the cell nuclei. Fluorescence was detected using an FV-1000 laser scanning inverted confocal microscope (Olympus, Tokyo, Japan).

### Statistical analysis

The data of in vitro experiments are shown as the mean ± standard error (SEM) from three or more independent experiments. All animal experiments were performed with five or more mice per group. The statistical analysis was performed using an unpaired two-tailed Student’s *t* test or the Mann‒Whitney test. One-way analysis of variance (ANOVA) with Tukey’s post hoc test was performed for comparisons among multiple experimental groups. Statistical analysis (*p < 0.05; and **p < 0.01) was carried out by using Prism version 7.0 (GraphPad, San Diego, CA).

## Results

### Changes in the population of IL-10^+^ ILCs and effector T cells in mice with CHS

**Fig. 1:**
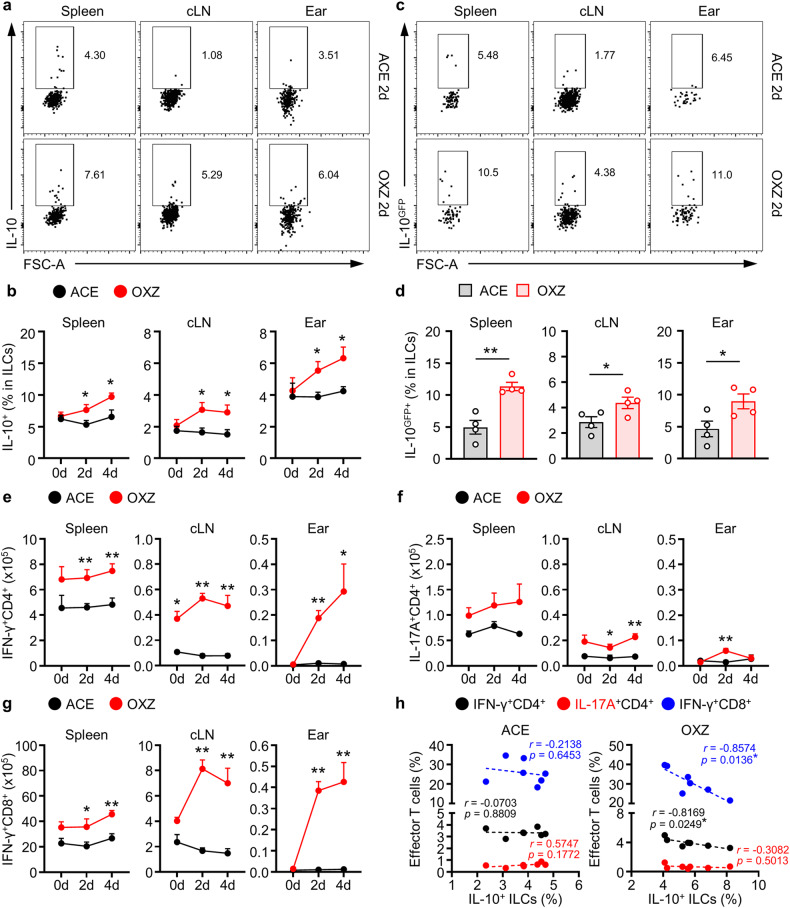
The population of the IL-10^+^ ILC subset increased in mice with OXZ-induced CHS. **a** Two days after OXZ challenge, representative plot images showing IL-10^+^ ILCs in the spleen, cLN and ear from B6 mice were created. **b** The histograms show the frequencies of IL-10^+^ ILCs in the spleen, cLN and ear at 0, 2, and 4 days after challenge with OXZ (*n* = 6). **c** Representative images of IL-10^GFP+^ ILCs in the spleen, cLN and ear from OXZ-induced *tiger* mice. The results are expressed as representative images (**a**) and the mean ± SEM (**b**) from at least three independent experiments (*n* = 2 per group for each experiment). **d** The histograms show the frequencies of IL-10^GFP+^ ILCs in the spleen, cLN and ear 2 days after challenge with OXZ (*n* = 4). The results are expressed as the mean ± SEM from two independent experiments (*n* = 2 per group for each experiment). *p < 0.05 and **p < 0.01 versus the ACE group according to Student’s *t* test. **e**–**g** The numbers of IFN-γ^+^CD4^+^ Th1 cells (**e**), IL^-^17A^+^CD4^+^ Th17 c**e**lls (**f**) and IFN-γ ^+^CD8^+^ CTL cells (**g**) during CHS are shown (n = 6). The results are expressed as the mean ± SEM from three independent experiments (*n* = 2 per group for each experiment). *p < 0.05 and **p < 0.01 versus the ACE group according to Student’s *t* test. **h** The correlation between the frequency of effector T-cell subsets and the frequency of IL-10^+^ ILCs in the spleen (*n* = 7). The p values were calculated by Pearson’s correlation coefficient. *p < 0.05.

Contact hypersensitivity is a well-known type of T-cell-mediated skin inflammation^25,26^. To analyze the alterations in IL-10^+^ ILCs and effector T cells in CHS, we induced CHS with OXZ in mice, and the cell population was subjected to individual analysis of the spleen, cervical lymph node (cLN), and ear tissue. The frequency of IL-10^+^ ILCs in the spleen, cLN and ear increased significantly in mice with CHS (Fig. 1a, b); this result was further validated in IL-10-GFP reporter mice (Fig. 1c, d). We also analyzed the changes in effector T cells, including Th1, Th17, and cytotoxic T cells, whose activation is critical for the induction of CHS^27–29^. The numbers of these effector T cells increased as CHS progressed (Fig. 1e–g). Interestingly, we further observed that there was an inverse relationship between the number of IL-10^+^ ILCs and the number of IFN-γ^+^CD4^+^ T cells or IFN-γ^+^CD8^+^ T cells in each mouse (Fig. 1h). These results led us to study whether IL-10^+^ ILCs could regulate the activity of effector T cells in mice with CHS.

### Adoptive transfer of IL-10^+^ ILCs suppresses CHS in mice

**Fig. 2:**
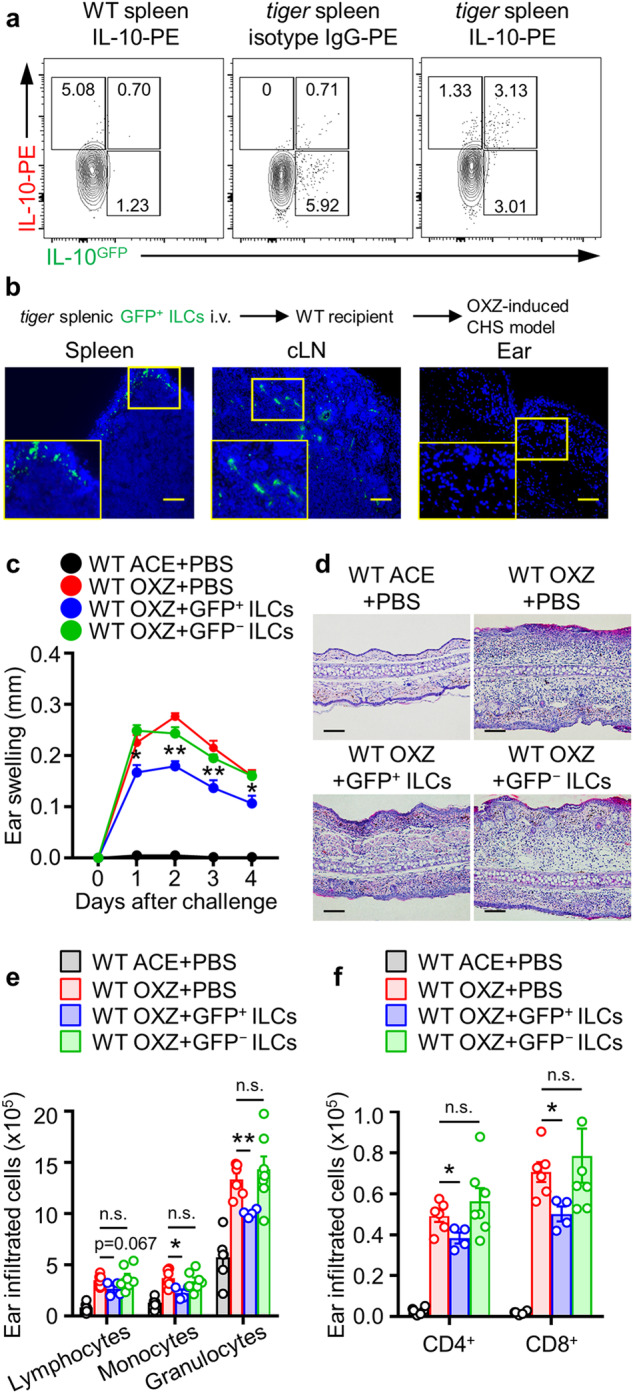
IL-10^GFP+^ ILCs ameliorate OXZ-induced CHS. **a** Representative flow cytometry images of IL-10^+^ and/or GFP^+^ ILCs from the spleens of WT or *tiger* mice. Lin^−^CD45^+^CD127^+^ ILCs were gated for IL-10 versus GFP expression. **b** GFP^+^ ILCs were isolated from *tiger* mice and i.v. transferred into WT mice (−1 day before sensitization). Four days after OXZ challenge, immunofluorescence staining was performed to detect infiltrated GFP^+^ ILCs in the spleen, cLN, and ear tissues by using an antibody against GFP (green). Scale bar, 100 μm. The results are presented as representative images from three independent experiments (**a**, **b**) (*n* = 2 per group for each experiment). **c**, **d** Ear thickness (**c**) and representative H&E image (**d**) of the ears of WT CHS mice after the transfer of GFP^+^ or GFP^−^ ILCs (*n* = 6). The data are presented as representative images (**c**) and the mean ± SEM (**d**) from two independent experiments (*n* = 3 per group for each experiment). *p < 0.05 and **p < 0.01 versus the WT OXZ + PBS-treated group according to Student’s *t* test. **e**, **f** Histograms showing the numbers of infiltrating lymphocytes, monocytes, granulocytes (**e**), CD4^+^ T cells, and CD8^+^ T cells (**f**) in the ear (*n* = 4–6) are shown. The data are expressed as the mean ± SEM from two independent experiments (*n* ≥ 2 per group for each experiment). *p < 0.05, **p < 0.01, and n.s., not significant versus the WT OXZ + PBS-treated group according to Student’s *t* test.

CHS is a typical type 1 helper T-cell-mediated skin inflammatory disease^26,28^; we attempted to determine whether IL-10^+^ ILCs suppress CHS and effector T cells in mice. The presence of GFP^+^ (as IL-10^+^) ILCs was detected in the spleens of *tiger* mice (Fig. 2a). Subsequently, we observed that the adoptively transferred GFP^+^ ILCs migrated to the spleen and LNs in normal mice, but these cells were not found in ear tissues (Fig. 2b). Notably, intravenous adoptive transfer of GFP^+^ ILCs, but not GFP^–^ ILCs, led to the suppression of ear swelling in mice with CHS (Fig. 2c, d). Repeatedly, the infiltration of most leukocytes (lymphocytes, monocytes, granulocytes, CD4^+^ T cells, and CD8^+^ T cells) into ear tissues was markedly inhibited by the adoptive transfer of GFP^+^ ILCs, but not GFP^−^ ILCs, into mice (Fig. 2e, f). We confirmed that GFP^+^ ILCs existed in the ear and underwent a change due to CHS induction (Fig. 1c, d), but due to logistical limitations, intravenously injected GFP^+^ ILCs were unable to directly migrate to the target site of CHS, which is the ear (Fig. 2b). Therefore, we sought to investigate the impact of direct intradermal injection of peripherally transferred GFP^+^ ILCs on CHS. Intradermally injected GFP^+^ ILCs also ameliorated the symptoms of CHS. While their impact on peripheral IL-17A^+^CD4^+^ T cells was modest, these cells demonstrated the capacity to suppress the activity of IFN-γ^+^CD4^+^ and CD8^+^ T cells (Supplementary Fig. 1). Collectively, these findings suggest that IL-10^+^ ILCs act as suppressors of CHS in mice.

### Sca-1 and PD-L1 expression in IL-10^+^ ILCs is dominant, and PD-L1^high^Sca-1^+^ ILCs are part of the ILC2_10_ subset

**Fig. 3:**
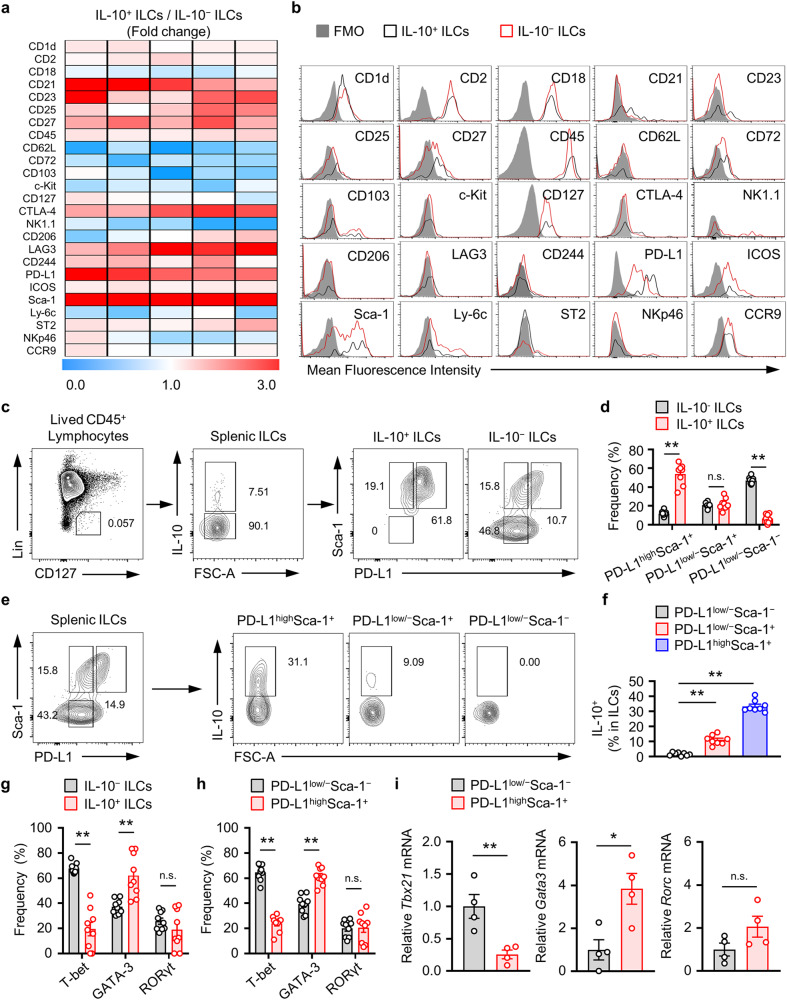
PD-L1^high^Sca-1^+^ cells are a novel IL-10-producing splenic ILC subset. **a** Surface marker protein expression profiling of the ratio of IL-10^+^ and IL-10^−^ from splenic ILCs. Heatmap depicting the ratio of IL-10^+^/IL-10^−^ ILCs obtained by flow cytometric analysis. **b** Representative flow cytometry images of each surface marker on IL-10^+^ versus IL-10^−^ ILCs. The results are presented as representative images from five independent experiments (**a**, **b**). **c**, **d** Representative plot images (**c**) and histograms (**d**) showing PD-L1^high^Sca1^+^, PD-L1^low/−^Sca-1^+^, and PD-L1^low/−^Sca-1^−^ subsets of IL-10^+^ and IL-10^−^ splenic ILCs (*n* = 8) from four independent experiments (*n* = 2 per group for each experiment). **p < 0.01, and n.s., not significant according to Student’s *t* test. **e**, **f** Representative plot images (**e**) and histograms (**f**) showing the frequency of IL-10^+^ ILCs among PD-L1^high^Sca1^+^, PD-L1^low/−^Sca-1^+^, and PD-L1^low/−^Sca-1^−^ ILC subsets (*n* = 8) from four independent experiments (*n* = 2 per group for each experiment). **p < 0.01 versus the PD-L1^low/−^Sca-1^−^ group according to Student’s *t* test. **g**, **h** Relative mean fluorescence intensities (MFIs) of T-bet, GATA-3 and RORγt in the indicated ILC subsets (three independent experiments; *n* = 3 per group for each experiment). **p < 0.01 and n.s., not significant versus the IL-10^−^ or PD-L1^low/−^Sca-1^−^ group according to Student’s *t* test. **i** The histograms show the mRNA expression levels of *Tbx21, Gata3* and *Rorc* in the PD-L1^high^Sca1^+^ and PD-L1^low/−^Sca-1^−^ ILC subsets (*n* = 4). The data are expressed as the mean ± SEM from four independent experiments. *p < 0.05, **p < 0.01, and n.s., not significant versus the PD-L1^low/−^Sca-1^−^ group according to Student’s *t* test.

Recently, Wang et al. highlighted the unique expression of the transcription factor Id3 in intestinal IL-10^+^ ILCregs, which distinguished these cells from other ILC subsets and regulatory T cells^20^. Other studies reported higher expression levels of the Id3, Foxf1, Atf3, and Klf2 genes in cultured ILC2 lineage IL-10-producing ILC2_10_s compared to that in typical activated ILC2s^21^. However, the surface markers of ILC2_10_s have not yet been identified. We previously reported the presence of the IL-10^+^ ILC subset in the spleen, lymph nodes, and ear tissue in mice, demonstrating that the expression of Sca-1, a typical ILC2 marker, was greater in IL-10^+^ ILCs than in IL-10^−^ ILCs^19^. In this study, we confirmed that the expression of Sca-1 was increased in IL-10^+^ ILCs compared to that in IL-10^−^ ILCs. In addition, we observed elevated expression of PD-L1, CTLA-1, CD21, and CD45 in IL-10^+^ ILCs compared to that in IL-10^−^ ILCs (Fig. 3a, b). Surprisingly, we found that splenic IL-10^+^ ILCs were largely included in the PD-L1^high^Sca-1^+^ ILC subset (Fig. 3c, d). Approximately 30% of the splenic PD-L1^high^Sca-1^+^ ILC subset was composed of IL-10^+^ ILCs, indicating a significant distribution of IL-10^+^ ILCs in this subset in comparison to the PD-L1^low/−^Sca-1^+^ or PD-L1^low/−^Sca-1^−^ ILC subset (Fig. 3e, f). IL-10^+^ ILCs, also known as ILC2_10_s_,_ are derived from ILC2 cells^21^. Next, we evaluated whether splenic IL-10^+^ ILCs are originated from ILC2_10_s and demonstrated the overwhelming frequency of GATA-3 expression in IL-10^+^ ILCs and PD-L1^high^Sca-1^+^ ILCs compared to that in T-bet and RORγt cells (Fig. 3g, h); GATA-3 is a typical transcription factor for ILC2s. Furthermore, compared with that in PD-L1^low/−^Sca-1^−^ ILCs, the amount of GATA-3 mRNA in PD-L1^high^Sca-1^+^ ILCs was significantly increased (Fig. 3i).

In a recent study by Wang et al., intestinal IL-10^+^ ILCregs were shown to produce TGF-β1, which is necessary for the expansion and survival of IL-10^+^ ILCregs^20^. In our study, we also examined the expression of TGF-β1 in splenic IL-10^+^ ILCs and PD-L1^high^Sca-1^+^ ILC subsets to investigate whether autocrine TGF-β1 is involved in the expansion of these subsets. While the expression of TGF-β1 may vary depending on the characteristics of tissue-specific ILCs, our study showed that the expression of TGF-β1 in splenic IL-10^+^ or PD-L1^high^Sca-1^+^ ILCs was extremely low (Supplementary Fig. 2). Taken together, these data suggest that the splenic IL-10^+^ and PD-L1^high^Sca-1^+^ ILC subsets may belong to the ILC2_10_ subset rather than the ILCreg population.

### Tissue distribution of the PD-L1^high^Sca-1^+^ ILC subset in mice

**Fig. 4:**
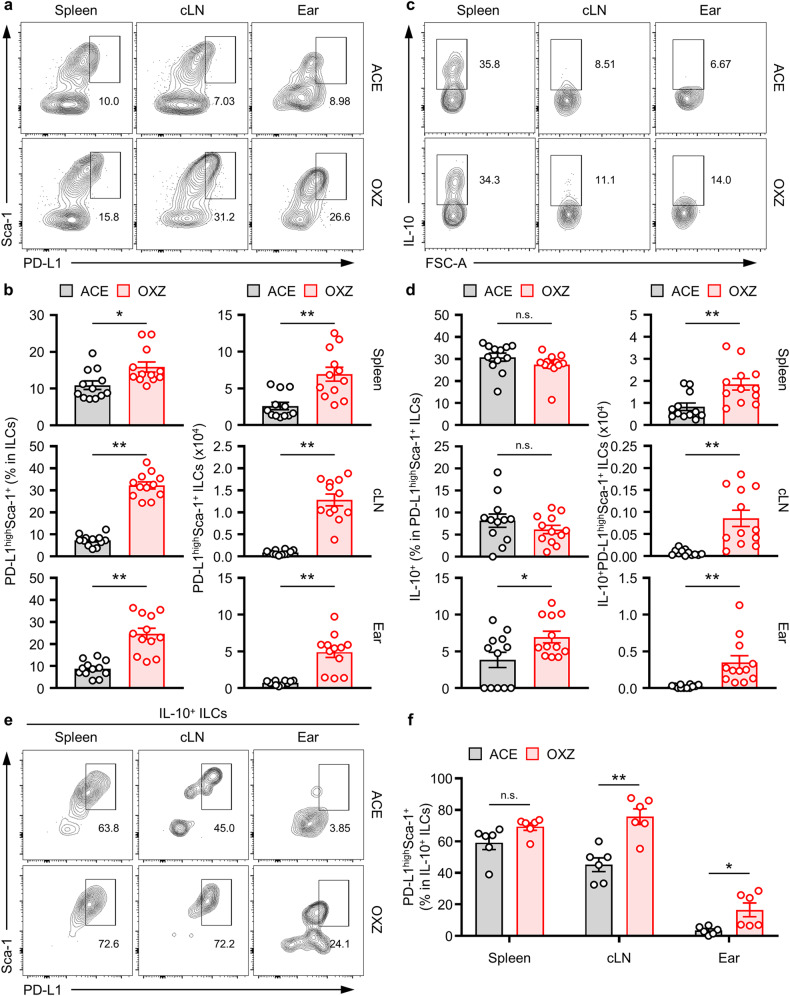
Population of IL-10-producing PD-L1^high^Sca-1^+^ ILCs in contact hypersensitivity. **a**, **b** Representative flow cytometry images (**a**) and histograms (**b**) of PD-L1^high^Sca-1^+^ ILC subsets in the spleen, cLN, and ears of mice with or without CHS (*n* = 12). **c**, **d** Representative flow cytometry images (**c**) and histograms (**d**) showing the frequency and number of IL-10^+^ ILCs in PD-L1^high^Sca-1^+^ ILC subsets from the spleen, cLN, and ear tissues (*n* = 12). The data are presented as representative images (**a**, **c**) of the mean ± SEM (**b**, **d**) from four independent experiments (n = 3 per group for each experiment). *p < 0.05 and **p < 0.01 versus the ACE group according to Student’s *t* test. **e,** **f** Represen*t*ative images (**e**) and histograms (**f**) of the frequencies of PD-L1^high^Sca-1^+^ ILC subsets among IL^−^10^+^ ILCs from the spleen, cLN, and ear in mice (*n* = 6) from two independent experiments (*n* = 3 per group for each experiment). *p < 0.05 and **p < 0.01 versus the ACE group according to Student’s *t* test.

We detected the presence of ILC2_10_s in the spleen, cLN, and ear of mice (Fig. 1a, c) and observed an increase in ILC2_10_s in each tissue as CHS symptoms progressed (Fig. 1b, d). Notably, a higher concentration of ILC2_10_s was found within the PD-L1^high^Sca-1^+^ ILC subset in the spleen (Fig. 3). Subsequently, we observed a remarkable increase in the PD-L1^high^Sca-1^+^ ILC subset in the spleen, cLN, and ear tissue in CHS mice compared to that in normal mice (Fig. 4a, b). Although the frequency of IL-10 expression in the PD-L1^high^Sca-1^+^ ILC subset remained similar, the number of PD-L1^high^Sca-1^+^ ILC subsets in each tissue significantly increased (Fig. 4c, d). Furthermore, our observation revealed that the proportion of ILC2_10_s with the PD-L1^high^Sca-1^+^ phenotype increased from 59.2 ± 4.3% to 69.4 ± 2.3% in the spleen when CHS was induced (Fig. 4e, f). In the cLN and ear, the frequencies changed from 45.1 ± 4.4% (cLN) and 3.4 ± 1.0% (ear) in the resting condition to 75.7 ± 4.9% (cLN) and 16.5 ± 4.4% (ear) in the CHS condition (Fig. 4e, f). These results suggested that ILC2_10_s are likely associated with CHS pathology in mice.

### IL-27 stimulates the development of ILC2_10_s and PD-L1^high^Sca-1^+^ ILC subsets

**Fig. 5:**
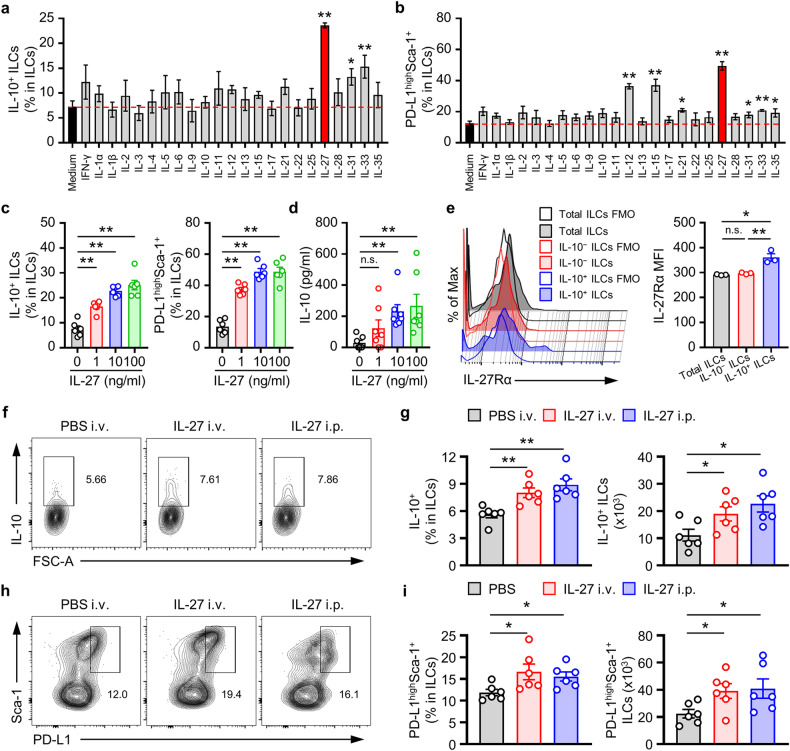
IL-27 stimulates the development of splenic IL-10-producing PD-L1^high^Sca-1^+^ ILCs. **a**, **b** Frequencies of the IL-10^+^ ILCs (**a**) and PD-L1^high^Sca-1^+^ ILC subsets (**b**) was determined via flow cytometry analysis of splenic Lin^−^ cells cultured for 12 h with the indicated cytokines (10 ng/ml). The data are presented as the mean ± SEM from four independent experiments. **c** Histograms showing the frequencies of IL-10^+^ ILCs and PD-L1^high^Sca-1^+^ ILC subsets of splenic Lin^−^ cells cultured for 12 h with recombinant IL-27 (1, 10, or 100 ng/ml) as indicated (*n* = 6). **d** The amount of IL-10 in culture media from Lin^−^ cells was determined via ELISA (*n* = 7). The results are expressed as the mean ± SEM from at least three independent experiments (**c**, **d**; *n* ≥ 2 per group for each experiment). *p < 0.05 and **p < 0.01 versus the untreated control of IL-27 according to Student’s *t* test. **e** The expression of IL-27Rα on the indicated ILC subsets was analyzed by flow cytometry. Representative plots (left) and mean fluorescence intensity (MFI, right) values are expressed as the mean ± SEM (right) from at least three independent experiments. *p < 0.05, **p < 0.01, and n.s., not significant according to one-way ANOVA with post hoc Tukey’s test. **f**−**i** Five days after treatment with rmIL-27 in vivo, the isolated splenocytes were analyzed via flow cytometry. Representative plots of IL-10^+^ ILCs (**f**) or PD-L1^high^Sca-1^+^ ILC subsets (**h**) and histograms showing the frequency and numbers of IL-10^+^ ILCs (**g**) or PD-L1^high^Sca-1^+^ ILC subsets (**i**) are shown (*n* = 6). The data are expressed as the mean ± SEM (**g**, **i**) from two independent experiments (*n* = 3 per group for each experiment). *p < 0.05 and **p < 0.01 versus the PBS group according to Student’s *t* test.

Next, we sought to identify the cytokines responsible for inducing the development of the ILC2_10_ and PD-L1^high^Sca-1^+^ ILC subsets. This study revealed that IL-27, IL-31, and IL-33 increased the population of ILC2_10_s; among these cytokines, IL-27 exhibited the most pronounced efficacy in inducing the development of ILC2_10_s (Fig. 5a). Recent studies have shown that the production of IL-10 by intestinal ILCregs and ILC2s markedly increased following stimulation with IL-2, TGF-β1, IL-4, IL-10, IL-27, or the neuromedin U^20,23^. However, unlike intestinal ILCregs and ILC2s, ILCs2_10_s were not induced from splenic ILCs by IL-2, IL-4, or IL-10 stimulation (Fig. 5a). Moreover, we observed a significant increase in the PD-L1^high^Sca-1^+^ ILC subset in response to IL-27 (Fig. 5b). Subsequent investigations revealed that IL-27 stimulated the formation of ILC2_10_ and PD-L1^high^Sca-1^+^ ILC subsets (Fig. 5c) but also markedly increased the secretion of IL-10 in a dose-dependent manner (Fig. 5d).

Recent Treg-related studies have highlighted the role of the autocrine IL-10 pathway in the expansion of IL-10-producing Tregs^30^. To verify whether the upregulation of IL-10 expression by IL-27 assumes a broader role beyond its regulatory function as an initiating mediator for regulatory subsets, we investigated the expression of the IL-10 receptor (IL-10R) on ILCs following exposure to IL-27. However, our assessment of IL-27-induced changes in IL-10R expression in ILCs via the autocrine IL-10 pathway did not reveal any IL-27-dependent alterations (Supplementary Fig. 3a). To enhance our understanding, we conducted in vitro experiments utilizing both WT and IL-10-deficient ILCs. Our aim was to analyze the impact of IL-27 on PD-L1^high^Sca-1^+^ ILC subsets and evaluate the extent of expansion in these subsets attributed to IL-10 deficiency. Under our experimental conditions, no significant differences were observed (Supplementary Fig. 3b). Thus, our findings suggest that the mechanism of IL-27-mediated activation of the IL-10^+^PD-L1^high^Sca-1^+^ ILC subset differs from the mechanism involving autocrine IL-10 production that occurs in Tregs.

Subsequent observations revealed that the expression of the IL-27 receptor on splenic ILC2_10_s was greater than that on the IL-10^−^ ILC subset (Fig. 5e), and a similar pattern was observed for the PD-L1^high^Sca-1^+^ ILC subset compared to the other subsets (Supplementary Fig. 4a, b). These results suggested that both the ILC2_10_ and PD-L1^high^Sca-1^+^ ILC subsets express the IL-27 receptor, indicating that IL-27 is likely required for ILC2_10_ induction and the subsequent secretion of IL-10. Furthermore, we administered IL-27 to mice to determine its effect on the development of splenic ILC2_10_s and PD-L1^high^Sca-1^+^ ILC subsets in vivo. Our results demonstrated that IL-27 administration increased the populations of the ILC2_10_ and PD-L1^high^Sca-1^+^ ILC subsets in mice (Fig. 5f–i). However, we did not observe significant changes in the expression of IL-10 in T cells, B cells, monocytes, or dendritic cells (Supplementary Fig. 5a). These results suggested that IL-27 plays a pivotal role as a critical cytokine in augmenting ILC2_10_ levels both in vitro and in vivo.

### IL-27 signaling is critical for the development of ILC2_10_s in mice

**Fig. 6:**
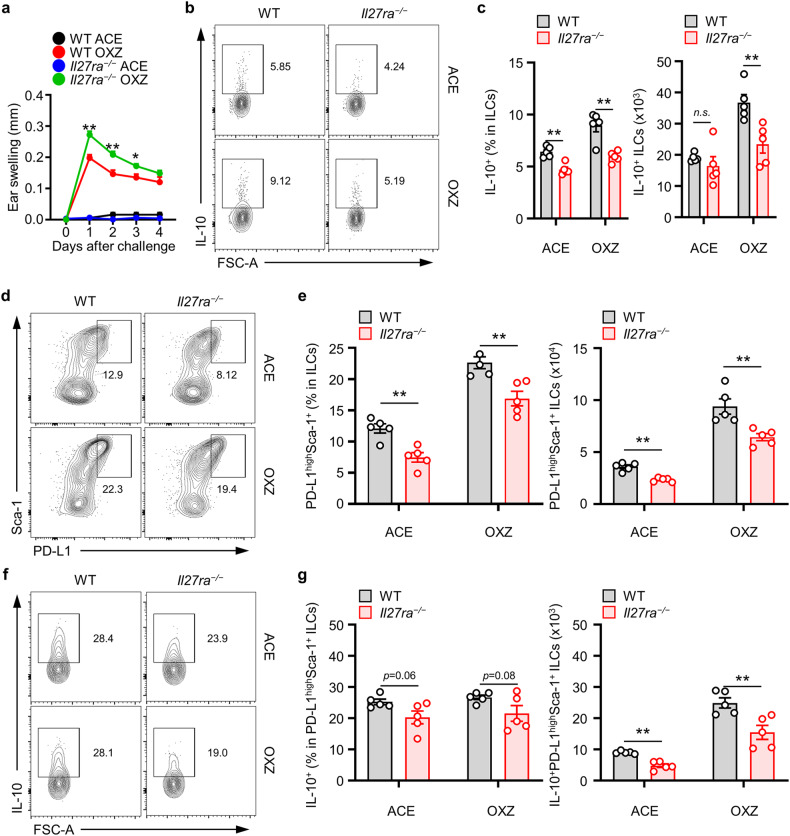
CHS is exacerbated by IL-27Rα deficiency. **a** Data of the ear thickness of WT or *Il27ra*^*−/*−^ mice 4 days after challenge with or without OXZ are shown. The data are expressed as the mean ± SEM from three independent experiments (*n* ≥ 3 per group for each experiment). *p < 0.05 and **p < 0.01 versus the WT OXZ group according to Student’s *t* test. **b** Representative plot images showing the number of IL-10^+^ ILCs in the spleen two days after OXZ challenge. **c** The histograms show the frequencies and numbers of IL-10^+^ ILCs in the spleen (*n* = 5). **d** Representative plots showing PD-L1^high^Sca-1^+^ ILCs in the spleens of WT or *Il27ra*^*−/−*^ mice. **e** Histograms showing th**e** frequencies and numbers of PD-L1^high^Sca-1^+^ ILCs in the spleens of WT or *Il27ra*^*−/−*^ mice (*n* = 5). **f** Representative plots showing IL-10^+^ ILCs among PD-L1^high^Sca-1^+^ ILCs from WT or *Il27ra*^*−/−*^ mice. **g** Histograms showing the frequencies and numbers of IL-10^+^ ILCs among PD-L1^high^Sca-1^+^ ILCs from WT or *Il27ra*^*−/−*^ mice (*n* = 5). The results are expressed as representative images (**b**, **d**, **e**) and the mean ± SEM (**c**, **e**, **g**). *p < 0.05 and **p < 0.01, and n.s., not significant versus WT mice according to Student’s *t* test.

Subsequent investigations revealed that the administration of IL-27 to mice led to the suppression of symptoms associated with CHS (Supplementary Fig. 5b). We further analyzed the ILC2_10_ and PD-L1^high^Sca-1^+^ ILC subsets following CHS induction in both WT mice and in those lacking the IL-27 receptor. The results demonstrated significantly more severe CHS symptoms in mice deficient in the IL-27 receptor than in WT mice (Fig. 6a). Additionally, our observations indicated a reduced frequency of ILC2_10_s in the spleens of IL-27-receptor-deficient mice than in WT mice (Fig. 6b, c). Furthermore, the absence of IL-27 signaling led to a decrease in the PD-L1^high^Sca-1^+^ ILC subset in the spleen (Fig. 6d, e), cLN, and ear (Supplementary Fig. 6) in mice. In the spleen, the number of IL-10^+^PD-L1^high^Sca-1^+^ ILCs also decreased (Fig. 6f, g). These results suggest that IL-27 signaling is pivotal for suppressing CHS symptoms by inducing ILC2_10_ development in peripheral tissues.

Antigen-presenting cells, such as macrophages and dendritic cells, are major sources of secreted IL-27^31–33^. To identify IL-27-producing cells in mice with CHS, we analyzed IL-27 expression in T cells, B cells, macrophages, monocytes, and dendritic cells. Consequently, the analysis led to the finding that macrophages are major IL-27-producing cells in the spleen, cLN, and ear in mice with CHS (Supplementary Fig. 7). Moreover, our observations highlighted the significant increase in IL-27 expression in macrophages as CHS progressed. IL-27 not only influences the maintenance of T-cell subsets but also participates in immune regulatory mechanisms by promoting the secretion of anti-inflammatory cytokines, such as IL-10^33,34,46^. Previous studies have reported the positive impact of IL-27 on T-cell activation via macrophages, which induced CHS^35^. In this context, we investigated the alterations in T-cell subset activation through IL-27 neutralization in CHS-induced mice. However, our findings did not reveal a statistically significant change in the distribution of T cells as a result of blocking the IL-27 mechanism during CHS induction (Supplementary Fig. 8). Overall, these findings suggest that IL-27 is associated with the induction of the ILC2_10_ population in mice with CHS.

### PD-L1^high^Sca-1^+^ ILC subsets in CHS suppress effector T cells in an IL-10-dependent manner

**Fig. 7:**
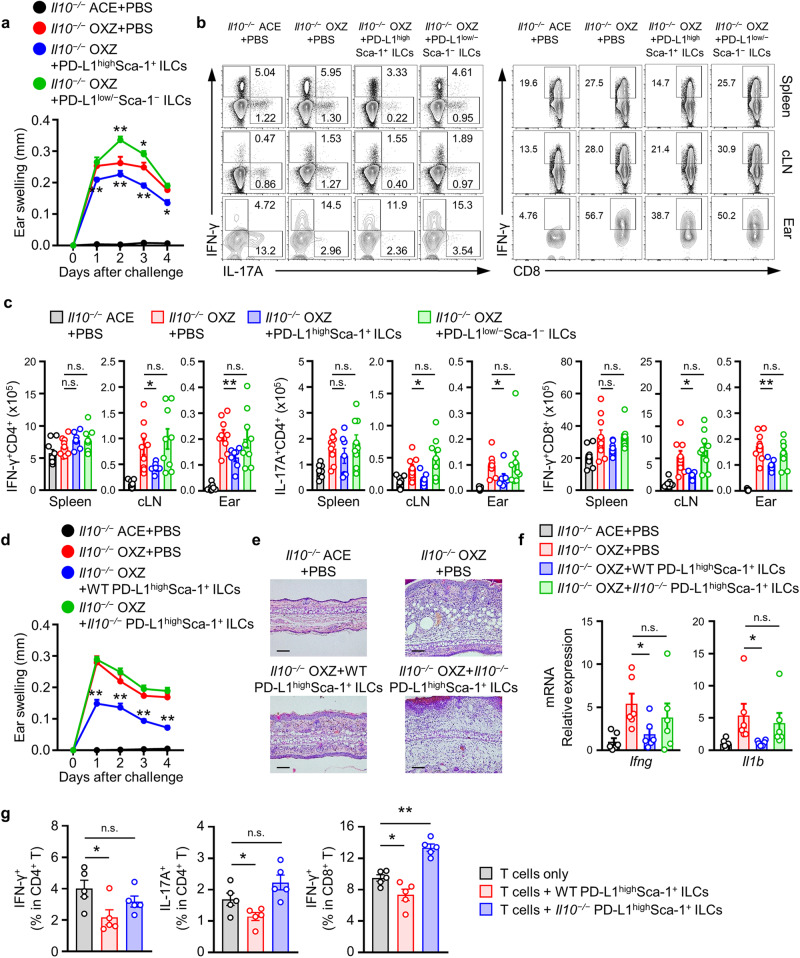
The PD-L1^high^Sca-1^+^ ILC subset suppresses CHS in an IL-10-dependent manner. **a** Data for the ear thickness of *Il10*^*−/−*^ mice with or without CHS are shown after the adoptive transfer of OXZ-sensitized WT PD-L1^high^Sca1^+^ or PD-L1^low/−^Sca-1^−^ ILC subsets, as indicated. The data are expressed as the mean ± SEM from three independent experiments (*n* ≥ 3 per group for each experiment). *p < 0.05 and **p < 0.01 versus the *Il10*^*−/−*^ OXZ + PBS group according to Student’s *t* test. **b**, **c** Represen*t*ative plots (**b**) and histograms (**c**) showing the numbers of IFN-γ^+^CD4^+^ Th1 cells, IL-17A^+^CD4^+^ Th17 cells, and IFN-^γ +^CD8^+^ CTL cells in the spleen, cLN, and ear (*n* = 7–9). The data are expressed as the mean ± SEM from three independent experiments (*n* ≥ 2 per group for each experiment). *p < 0.05, **p < 0.01, and n.s., not significant versus the *Il10*^*−/−*^ OXZ + PBS group according to Student’s *t* test. **d**, **e** Ear thickness (**d**) and represen*t*ative H&E image (**e**) of the ears of *Il10*^*−/−*^ mice with or without CHS after the transfer of WT or *Il10*^*−/−*^ PD-L1^high^Sca1^+^ ILCs. The data are expressed as the mean ± SEM from four independent experiments (*n* = 3 per group for each experiment). **p < 0.01 versus the *Il10*^*−/−*^ OXZ + PBS group according to Student’s *t* test. **f** The histograms show the mRNA expression levels of *Ifng* and *Il1b* in ear tissues from *Il10*^*−/−*^ mice after the transfer of WT or *Il10*^*−/−*^ PD-L1^high^Sca1^+^ ILCs (*n* = 6). The data are expressed as the mean ± SEM from two independent experiments (*n* = 3 per group for each experiment). *p < 0.05 and n.s., not significant versus the *Il10*^*−/−*^ OXZ + PBS group according to Student’s *t* test. **g** CD4^+^ T cells or CD8^+^ T cells from WT mice were cultured with PD-L1^high^Sca1^+^ ILCs derived from WT or *Il10*^*−/−*^ mice at a 2:1 ratio for 1 day. The histogram shows the frequencies of IFN-γ^+^/IL-17A^+^CD4^+^ T cells and IFN-γ^+^CD8^+^ T cells in vitro. The data are expressed as the mean ± SEM from five independent experiments. *p < 0.05, **p < 0.01, and n.s., not significant versus the only T cells group according to Student’s *t* test.

Currently, comprehensive information regarding the surface markers of ILC2_10_s is limited. Therefore, we tested the effect of controlling CHS following the adoptive transfer of PD-L1^hi^Sca-1^+^ ILCs into mice. Initially, we isolated the PD-L1^high^Sca-1^+^ ILC subset and PD-L1^low/−^Sca-1^−^ ILC subset using FACS Aria. In IL-10-deficient mice, adoptive transfer of the PD-L1^high^Sca-1^+^ ILC subset inhibited CHS symptoms, while the PD-L1^low/−^Sca-1^−^ ILC subset tended to slightly exacerbate these symptoms (Fig. 7a). Furthermore, our experiments demonstrated a reduction in the number of IFN-γ^+^CD4^+^ T cells, IL-17A^+^CD4^+^ T cells, and IFN-γ^+^CD8^+^ T cells in the cLN and ear following the adoptive transfer of the PD-L1^high^Sca-1^+^ ILC subset but not the PD-L1^low/−^Sca-1^−^ ILC subset (Fig. 7b, c). However, no significant changes were observed in Foxp3^+^ regulatory T cells in the spleen, cLN, or ear during the same experiment (Supplementary Fig. 9). Collectively, these results suggest that the PD-L1^high^Sca-1^+^ ILC subset suppresses CHS symptoms by inhibiting the activation of effector T cells.

Subsequently, we investigated whether the impact of the PD-L1^high^Sca-1^+^ ILC subset on CHS is dependent on IL-10. Notably, in IL-10-deficient mice, CHS symptoms and the infiltration of immune cells were inhibited by the WT PD-L1^high^Sca-1^+^ ILC subset but not by the IL-10-deficient PD-L1^high^Sca-1^+^ ILC subset (Fig. 7d, e). Additionally, adoptive transfer of the WT PD-L1^high^Sca-1^+^ ILC subset significantly suppressed the expression of *Ifng* and *Il1b* mRNA in ear tissue (Fig. 7f).

To further explore the in vitro regulation of CHS by PD-L1^high^Sca-1^+^ ILC subsets in an IL-10-dependent manner, we employed an in vitro coculture system. PD-L1^high^Sca-1^+^ ILC subsets were isolated from both WT and *Il10*^−*/*−^ mice and cultured with CD4^+^ or CD8^+^ T cells obtained from WT mice. Our observations revealed that the WT PD-L1^high^Sca-1^+^ ILC subset could modulate the expression of IFN-γ and IL-17A in CD4^+^ T cells. Conversely, the IL-10-deficient PD-L1^high^Sca-1^+^ ILC subset lacked this regulatory capacity. Similarly, we observed suppression of IFN-γ^+^CD8^+^ T-cell activity in cells cocultured with the WT PD-L1^high^Sca-1^+^ ILC subset. Intriguingly, the IL-10-deficient PD-L1^high^Sca-1^+^ ILC subset, on the other hand, induced an increase in IFN-γ expression in CD8^+^ T cells (Fig. 7g). Overall, our findings underscore the critical role of the PD-L1^high^Sca-1^+^ ILC subset, including ILC2_10_s, in suppressing CHS symptoms by inhibiting effector T cells in an IL-10-dependent manner.

## Discussion

ILCs play an important role in maintaining tissue homeostasis and peripheral immunity^1^. ILCs are generally classified in parallel as different types of helper T (Th) cells, such as Th1, Th2, and Th17 cells. Although not always definitive, the following definitions of the three typical types of ILC subsets are generally accepted: the T-bet-expressing and IFN-γ-secreting ILC subset is the ILC1 subset; the GATA3-expressing and Th2-cytokine subset includes IL-5, IL-9, and IL-13, and this is the ILC2 subset; and the RORγt-expressing ILC subset that produces IL-17A or IL-22 is referred to as the ILC3 subset^1,4,36^. Based on the knowledge that Foxp3^+^CD4^+^ T cells suppress various inflammatory immune responses, we wanted to determine whether such regulatory subsets also exist in ILCs. Recent reports have identified a unique ILC subset capable of producing IL-10^19^. Subsequently, Wang et al. reported a regulatory ILC subset that suppresses innate immune responses during intestinal inflammation in an IL-10-dependent manner^20^. Moreover, IL-10-producing ILCs are derived from the ILC2 subset and are often referred to as ILC2_10_s^21–23^. However, the precise classification of IL-10^+^ ILCs within established ILC subsets or as a distinct subset within ILCs remains subject to debate. However, the anti-inflammatory effects of all IL-10-producing ILC subsets, including ILCregs and ILC2_10_s, are dependent on IL-10. IL-10^+^ ILCs inhibit the activity of other ILC subsets or other leukocytes in peripheral tissues and restrain the proliferation of T cells in vitro^21–23^. In this study, we further revealed that PD-L1^high^Sca-1^+^ ILCs, including ILC2_10_s, inhibit OXZ-induced CHS symptoms, primarily by suppressing effector T cells in an IL-10-dependent manner.

CHS represents a well-established form of T-cell-mediated skin inflammation^37^. In our previous study, we highlighted an increase in the population of IL-10^+^ ILCs in OXZ-induced CHS mice; these results provided initial evidence for the existence of a regulatory ILC subset, as is found in T cells or B cells^19^. Interestingly, we observed an inverse correlation between the number of IFN-γ^+^CD4^+^ T cells and CD8^+^ T cells and the number of IL-10^+^ ILCs within individual spleens from mice with CHS (Fig. 1h). These findings present, for the first time, evidence that IL-10^+^ ILCs may suppress the functions of these effector T cells, thereby suppressing CHS symptoms in mice.

We further investigated the therapeutic potential of intravenously delivered IL-10^+^ ILCs in regulating CHS. Our findings indicated that intravenously administered IL-10^+^ (GFP^+^) ILCs effectively reached the spleen and cLN during the CHS induction phase, as shown in Fig. 2b. However, these ILCs did not reach the intended peripheral tissue, the ear. Nevertheless, administration of IL-10^+^ ILCs showed that the regulatory effects of IL-10^+^ ILCs may extend beyond peripheral tissues. This result was substantiated by a reduction in CHS-induced ear swelling and the inhibition of leukocyte infiltration into ear tissue (Fig. 2c–f). Notably, peripherally introduced IL-10^+^ ILCs also exhibited the capacity to suppress CHS lesions in ear tissue (Supplementary Fig. 1). Consequently, we anticipate that the CHS-dependent increase in the number of IL-10^+^ ILCs residing in lymphoid organs and peripheral tissues may have a significant impact on T-cell-mediated development of CHS.

Generally, diverse expression levels of cell surface marker proteins are utilized to characterize immune cells, including ILC subsets. While the surface phenotype of ILC1s shares similarities with that of NK cells or some ILC3s, they can be differentiated by distinct expression patterns, such as those of NK1.1 or NKp46, from NK cells or ILC3s^3,38^. Inducible T-cell costimulator (ICOS), chemoattractant receptor-homologous molecule expressed on Th2 cells (CRTH2) and killer-cell lectin like receptor G1 (KLRG1) are well-established surface proteins of ILC2s^39–41^. ILC3s encompass subclasses such as lymphoid tissue inducer cells (LTis), NKp46^+^ ILC3s, and NKp46^−^ ILC3s^42^. However, the surface phenotype of IL-10^+^ ILCs has not been characterized. To determine the unique phenotypic traits of splenic IL-10^+^ ILCs, we compared the expression patterns of molecules between IL-10^+^ ILCs and IL-10^−^ ILCs. Among several surface proteins, PD-L1 and Sca-1 were expressed at notably higher levels in IL-10^+^ ILCs than in IL-10^−^ ILCs (Fig. 3a). A substantial proportion of IL-10^+^ ILCs were PD-L1^high^Sca-1^+^ ILCs (53.86 ± 4.10%), and the proportion of PD-L1^high^Sca-1^+^ ILCs in IL-10^+^ ILCs significantly exceeded that of IL-10^−^ ILCs (11.97 ± 0.99%) (Fig. 3d). Notably, compared with PD-L1^low/−^Sca-1^−^ ILCs, splenic IL-10^+^ ILCs were markedly enriched in PD-L1^high^Sca-1^+^ ILCs (approx. 33%) (if any, minimal) (Fig. 3f). As the number of IL-10^+^ ILCs increased, there was a significant increase in the number of PD-L1^high^Sca-1^+^ ILC subsets in mice with CHS compared to that of mice without CHS (Fig. 4). Collectively, these findings indicate extensive enrichment of IL-10-producing ILCs within the PD-L1^high^Sca-1^+^ ILC subset.

In light of the findings of previous studies, IL-10^+^ ILCs have been identified as a subset referred to as ILC2_10_s. Our earlier observations revealed IL-10 expression in Lin^−^CD45^+^CD127^+^Sca-1^+^ILCs^19^. Given previous reports identifying Sca-1 as a surface marker of ILC2s, it was implied that IL-10 expression in ILCs might originate from IL-10^+^ ILC2s. Additionally, Corey et al. corroborated this finding by demonstrating an increase in IL-10 expression in ILC2s in the lung following in vivo injection of IL-33; they designated these IL-10-producing ILC2s as ILC2_10_s^21^. Jennifer et al. reported increased IL-10 expression in intestinal ILC2s when stimulated with IL-2, IL-4, IL-10, IL-27, and neuromedin U in vitro^23^. In addition, ILC2_10_s were identified in human PBMCs and nasal tissues^22,43^. Consistent with these findings, our study revealed overwhelming GATA3 expression in IL-10^+^ ILCs and PD-L-1^high^Sca-1^+^ ILCs (Fig. 3g–i). These results further substantiate the idea that the IL-10^+^ ILCs in our experimental setup are consistent with the characteristics of ILC2_10_s, as has been observed in previous reports. Furthermore, our findings revealed the presence of substantial amounts of ILC2_10_s within the PD-L1^high^Sca-1^+^ ILC population.

ILC subsets are present throughout the body, with varying distributions in various peripheral tissues^44^. In this study, we found that in normal mice, the PD-L1^high^Sca-1^+^ ILC subset represented approximately 10% of the total ILC population in the spleen, lymph nodes, and ear tissues. Furthermore, upon induction of CHS, these frequencies increased to approximately 15% in the spleen, 33% in the lymph nodes, and 23% in the ear tissues (Fig. 4b). Interestingly, while the frequency of IL-10^+^ ILCs within the PD-L1^high^Sca-1^+^ ILC subset remained stable in the spleen and lymph nodes (Fig. 4d), only a slight increase was observed in inflamed ear tissues. However, the overall number of IL-10^+^PD-L1^high^Sca-1^+^ ILCs was greater in CHS mice than in normal mice (Fig. 4b, d). These findings suggest a close correlation between the PD-L1^high^Sca-1^+^ ILC subset and the suppression of CHS symptoms through IL-10 production.

Thus far, various molecules, including TGF-β1, IL-2, IL-4, IL-27, IL-10, IL-33, neuromedin U, and retinoic acid, have been shown to activate IL-10-producing ILCs^20–23^. In this study, we measured the effect of stimulating ILCs with diverse cytokines on the mutual increase in the number of IL-10^+^ ILCs and the PD-L1^high^Sca-1^+^ ILC subset in vitro. Among the various cytokines tested, IL-27 was found to be the most effective at promoting the expansion of IL-10^+^ ILCs and the PD-L1^high^Sca-1^+^ ILC subset (Fig. 5a–d). These results were also supported by the results obtained after induction with IL-27 in vivo (Fig. 5f–i). Recent studies have reported the ability of IL-2, IL-4, and IL-33 to induce IL-10 production in ILCs^20–23^, and our study also revealed a partial increase in response. However, conditions satisfying both IL-10 production and an increase in PD-L1^high^Sca-1^+^ ILC subsets were uniquely observed with IL-27. The variability in these outcomes might be influenced by the characteristics of the tissue-specific ILCs used, variations in associated cell expression phenotypes, and the specific culture conditions.

IL-27, which is a member of the IL-12 cytokine family that is composed of the IL-27p28 and Ebi3 subunits, is produced by antigen-presenting cells (APCs)^33^. The functions of Il-27 include the induction of Th1 cell expansion by activating the T-bet-dependent pathway and its pivotal role in suppressing Th17-mediated inflammation^45,46^. In a distinct CHS model triggered by DNCB, myeloid cells in peripheral tissues, which secrete IL-27, were shown to play a vital role by inducing IL-15 production by keratinocytes, thereby contributing to the maintenance of CD8^+^ T cells. This finding highlights the role of IL-27 as an effector cytokine^35^. Moreover, IL-27 has capacity to induce IL-10 production from lymphocytes^47–49^. In our study, the administration of IL-27 to mice led to the suppression of OXZ-induced CHS symptoms (Supplementary Fig. 5a). In mice lacking the IL-27 receptor, we further observed suppression of the splenic IL-10 + ILC population (Fig. 6c) and significant exacerbation of CHS symptoms (Fig. 6a). However, our in vivo IL-27p28 mAb neutralization experiments did not reveal significant changes in CD4^+^ or CD8^+^ T-cell subsets within the spleen, cLN, or peripheral ear tissues. These findings collectively suggest the existence of a unique regulatory axis for IL10^+^ ILCs mediated by IL-27; thus, IL-27 contributed to the preservation of peripheral tolerance in the OXZ-induced CHS model.

IL-10 plays a pivotal role in suppressing the immune responses of effector T cells and inhibiting the activation of APCs and myeloid lineage cells; this role includes the suppression of MHC or B7 expression and the production of pro-inflammatory cytokines^50,51^. IL-10 is secreted by diverse leukocytes, such as T cells, B cells, NK cells, monocytes, macrophages, and dendritic cells^52–55^. This finding prompted us to investigate whether the regulatory function of PD-L1^high^Sca-1^+^ ILCs is IL-10 dependent. The CHS response was significantly suppressed by the adoptive transfer of PD-L1^high^Sca-1^+^ ILCs, whereas neither PD-L1^low/−^Sca-1^−^ ILCs nor IL-10-deficient PD-L1^high^Sca-1^+^ ILCs exhibited a similar suppressive effect (Fig. 7a, d).

Furthermore, i.v. transfer of WT PD-L1^high^Sca-1^+^ ILCs into IL-10-deficient mice reduced the number of effector T cells, including Th1, Th17, and cytotoxic T cells, in the cLNs and ear tissues (Fig. 7b, c). Notably, PD-L1 impacts immune responses as a ligand of the PD-1 receptor that inhibits effector T cells^56^. Therefore, exploring whether PD-L1 expression in PD-L1^high^Sca-1^+^ ILCs is associated with the inhibition of CHS is worthwhile. A comparison of the adoptive transfer of WT or IL-10-deficient PD-L1^high^Sca-1^+^ ILCs into CHS mice revealed that IL-10-deficient PD-L1^high^Sca-1^+^ ILCs did not exert a suppressive effect (Fig. 7d). These findings imply that the inhibitory effect of PD-L1^high^Sca-1^+^ ILCs on CHS is dependent on IL-10 but not PD-L1.

In diverse studies exploring the IL-10-mediated immune regulatory system, the interplay among regulatory cells has been increasingly studied. Reports have highlighted an additional regulatory mechanism involving IL-10-producing cells, such as Tr1 cells or ILCregs, which influence the maintenance of IL-10^+^ regulatory cell function and immune regulatory cell proliferation via autocrine secretion of TGF-β1 or IL-10^20,30^. Our investigation of the IL-10-deficient CHS mouse model validated the regulatory effects of the IL-10^+^PD-L1^high^Sca-1^+^ ILC subset (Fig. 7), thereby establishing an independent regulatory mechanism not contingent upon Tregs (Supplementary Fig. 9). Furthermore, our observations revealed lower levels of TGF-β1 secretion in the IL-10^+^ or PD-L1^high^Sca-1^+^ ILC subsets than in the other subsets (Supplementary Fig. 2), and the proliferation of the IL-10^+^PD-L1^high^Sca-1^+^ ILC subsets induced by IL-27 was shown to be independent of secreted IL-10 (Supplementary Fig. 3). Consequently, within the CHS mouse model, no specific additional mechanisms mediated by autocrine TGF-β1 or IL-10 were identified within the immune regulatory mechanism of the IL-27 and IL-10^+^PD-L1^high^Sca-1^+^ ILC subsets.

We noted a systemic increase in the number of IL-10^+^ ILCs following CHS induction, with the spleen emerging as the primary reservoir of these cells (Fig. 1b, d). Intravenous administration of splenic IL-10^+^ (GFP^+^) ILCs did not lead to their localization at CHS target sites, such as the ear; rather, their accumulation occurred in the spleen and cLN (Fig. 2b). Intriguingly, this treatment ameliorated CHS symptoms (Fig. 2c) and hindered the migration of T cells to the ear (Fig. 2f). Furthermore, intravenous administration of PD-L1^high^Sca-1^+^ ILCs regulated effector T-cell activity in both the cLN and ear, and these effects were dependent on IL-10 (Fig. 7c). Subcutaneous administration of splenic IL-10^+^ (GFP^+^) ILCs also significantly alleviated disease (Supplementary Fig. 1). However, these splenic IL-10^+^ ILCs are presumed to primarily control the propagation of peripheral inflammation originating from secondary lymphoid organs, including the spleen and cLN.

While our investigation underscores the significance of splenic IL-10^+^ ILCs based on their systemic distribution ratio, it is possible that IL-10^+^ ILCs within draining lymph nodes play a crucial role in regulating inflammatory cell activity, especially that of effector T cells, from an early disease pathogenesis perspective. In summary, this study yields pioneering findings demonstrating the critical role of the IL-27-induced IL-10-producing splenic PD-L1^high^Sca-1^+^ ILC subset in maintaining immune homeostasis in skin.

### Supplementary information

The online version of the manuscript contains supplementary material available at Correspondence and requests for materials should be addressed to Hyuk Soon Kim or Wahn Soo Choi.

### Supplementary information

Supplementary Information
