# Supplementary material for: IL-27-induced PD-L1highSca-1+ innate lymphoid cells suppress contact hypersensitivity in an IL-10-dependent manner
Source: Exp Mol Med. 2024 Mar 1;56(3):616–29. doi: 10.1038/s12276-024-01187-1 (PMC10984996; doi:10.1038/s12276-024-01187-1)
Supplement: Supplementary file 1 — Supplementary Information [file 12276_2024_1187_MOESM1_ESM.pdf]

Supplementary Information

IL-27-induced PD-L1^{high}Sca-1^{+} innate lymphoid cells suppress contact hypersensitivity in an IL-10-dependent manner

Keun Young Min,[1,†] Do-Kyun Kim,[2,†] Min Geun Jo,[1] Min Yeong Choi,[1] Dajeong Lee,[1] Jeong Won Park,[3] Young-Jun Park,[4] Yeonseok Chung,[4] Young Mi Kim,[5] Yeong-Min Park,[1] Hyuk Soon Kim,[3,*] and Wahn Soo Choi,[1,*]

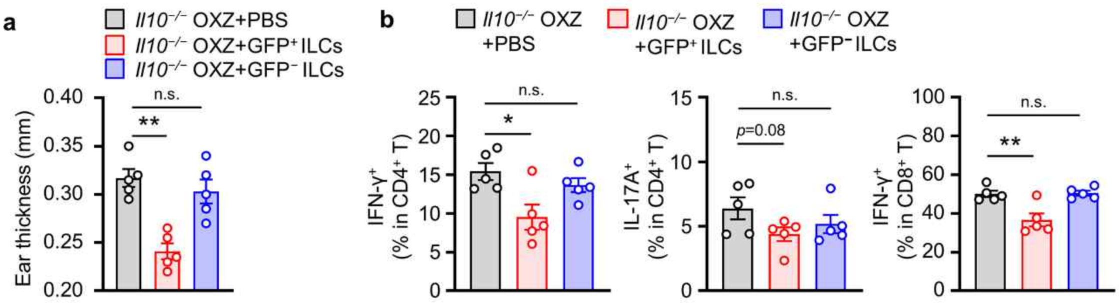

Supplementary Fig 1. Effects of intradermally transferred IL-10^{GFP+} ILCs in IL-10-deficient CHS mice. a IL-10^{GFP+} ILCs from *tiger* mice are intradermally transferred into *Il10*$^{-/-}$ mice one day before sensitization. The ear thickness is measured on 2 days after challenge. **b** Frequency of IFN-γ and IL-17A in CD4^{+} T cells and IFN-γ in CD8^{+} T cells in ear of the CHS mice (2 days post-OXZ challenge). The data are expressed as the mean ± SEM (**a**, **b**) from two independent experiments (n=2-3 per group for each experiment). *p < 0.05 and **p < 0.01 versus PBS-treated group by Student's *t*-test.

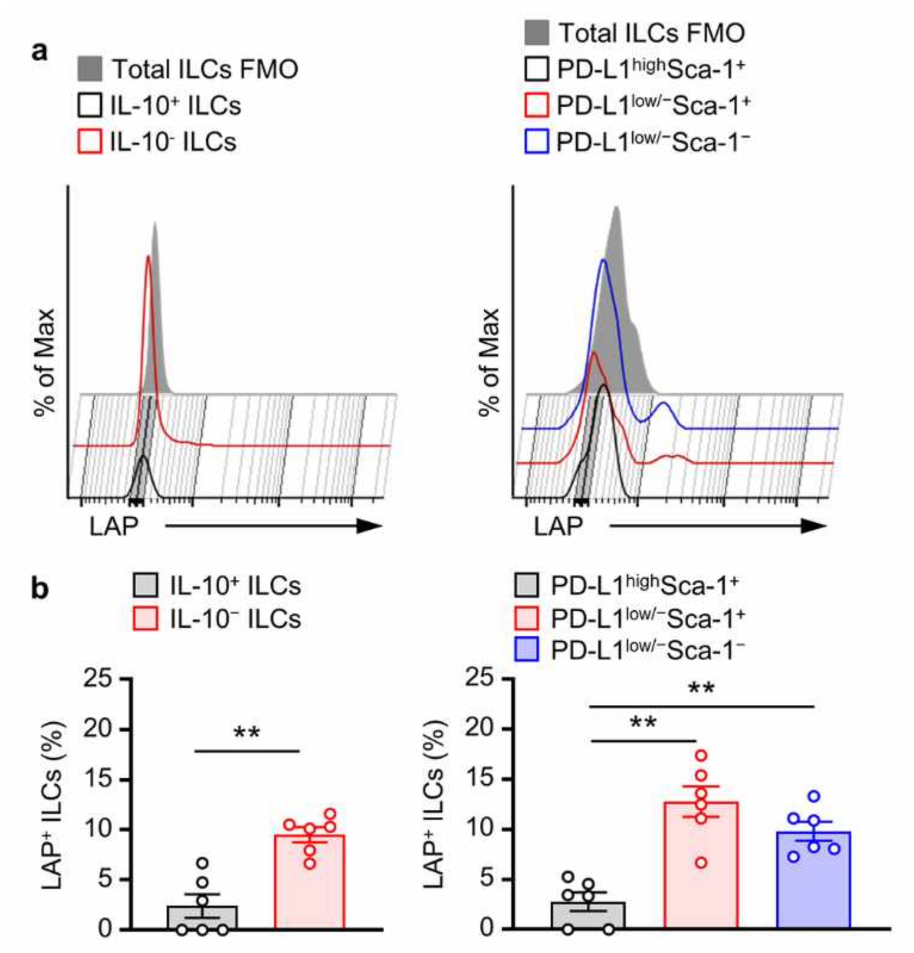

Supplementary Fig 2. The LAP expression of IL-10$^+$ ILCs and PD-L1^{high}Sca-1$^+$ ILCs. a Representative histograms show the frequencies of LAP (as TGF-β1) in IL-10$^+$ and IL-10$^-$ Splenic ILCs, or in PD-L1^{high}Sca1$^+$, PD-L1$^{low/-}$Sca-1$^+$, and PD-L1$^{low/-}$Sca-1$^-$ subsets. **b** The histograms show the frequencies of the LAP+ subsets on indicated populations. The data are expressed as the mean ± SEM from two independent experiments (n=3 per group for each experiment). **p < 0.01 by Student's *t*-test.

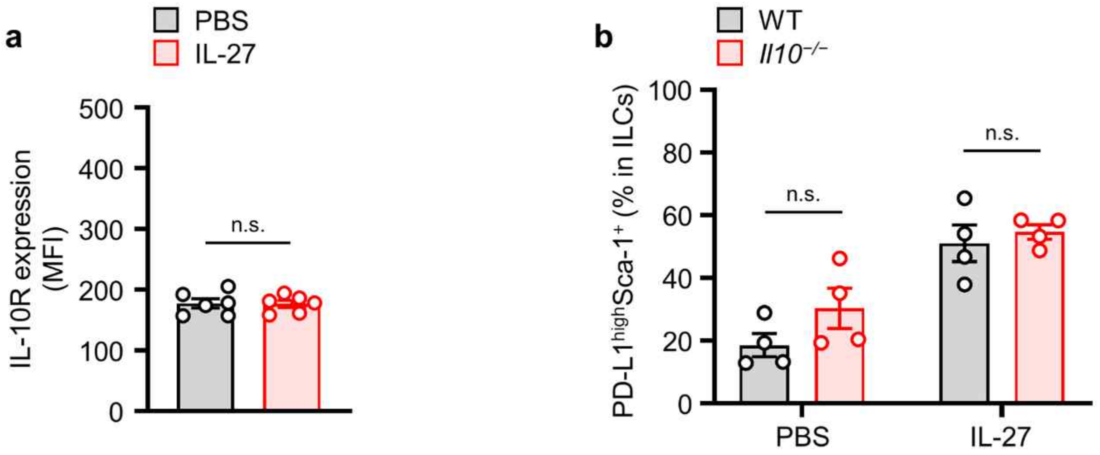

Supplementary Fig 3. Evaluation of the autocrine effect of IL-10 produced by IL-27 in ILCs. a The histograms show the expression of IL-10R (MFI) in PBS or IL-27-treated splenic ILCs (n=6). The data are expressed as the mean ± SEM from three independent experiments (n=2 per group for each experiment). n.s., not significant by Student's *t*-test. **b** The histograms show the frequencies of PD-L1^{high}Sca-1^{+} in PBS or IL-27-treated splenic ILCs from WT or in *Il10*$^{-/-}$ mice (n=4). The data are expressed as the mean ± SEM from four independent experiments. n.s., not significant by Student's *t*-test.

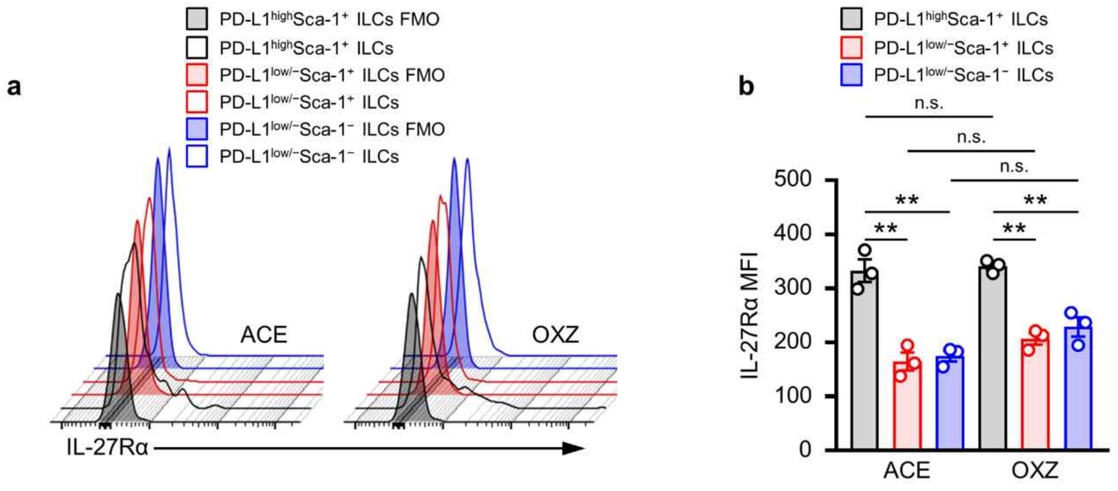

Supplementary Fig 4. IL-27Rα expression on PD-L1^{high}Sca-1^{+} ILC Subset. a The expression level of IL-27Rα on indicated ILC subsets was analyzed by flow cytometry. **b** The MFI values are expressed as the mean ± SEM from at least three independent experiments. **$p < 0.01$ and n.s., not significant by one-way ANOVA with post hoc Tukey's test.

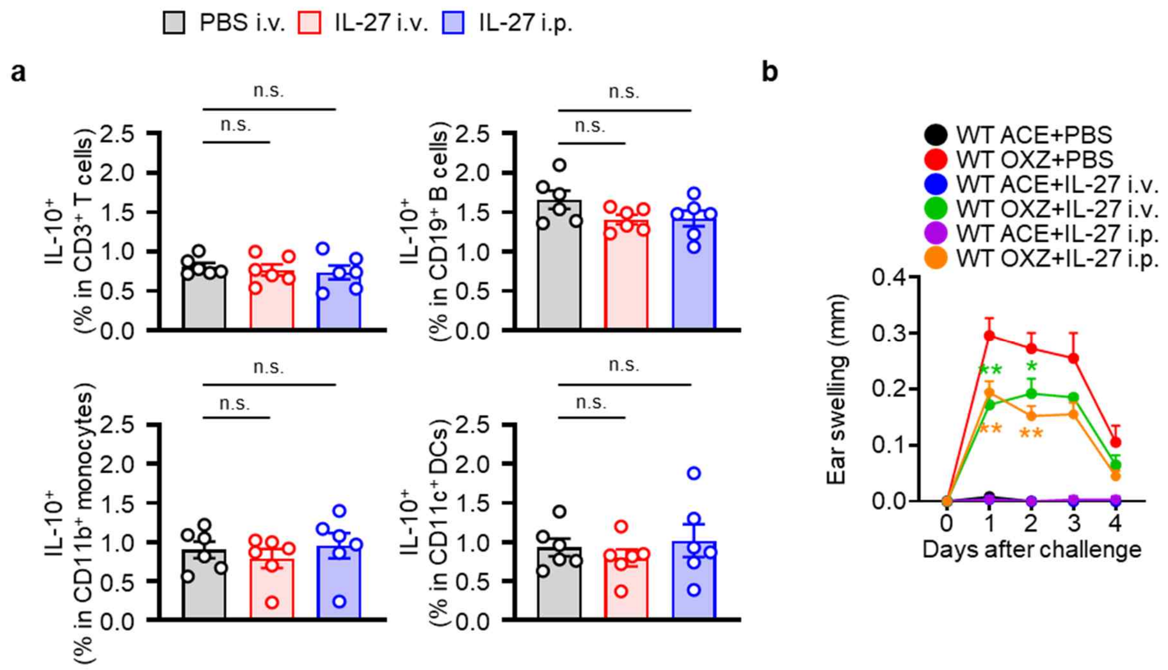

Supplementary Fig 5. rmIL-27 is not required for IL-10-producing other leukocytes activation in vivo and suppresses OXZ-induced CHS in mice. a Five days after rmIL-27 treatment in vivo, the histograms show the frequencies of IL-10$^+$ leukocytes (CD3$^+$ T cells, CD19$^+$ B cells, CD11b$^+$ monocytes and CD11c$^+$ dendritic cells) in spleen (n=6). The data are expressed as the mean ± SEM. n.s., not significant versus PBS-treated group by Student's *t*-test. **b** Ear thickness of WT CHS mice with rmIL-27 (i.v. or i.p.) treatment (n=10−12). The data are expressed as the mean ± SEM pooled from two independent experiments. (n≥3 per group for each experiment). *$p < 0.05$ and **$p < 0.01$ versus WT OXZ PBS-treated group by Student's *t*-test.

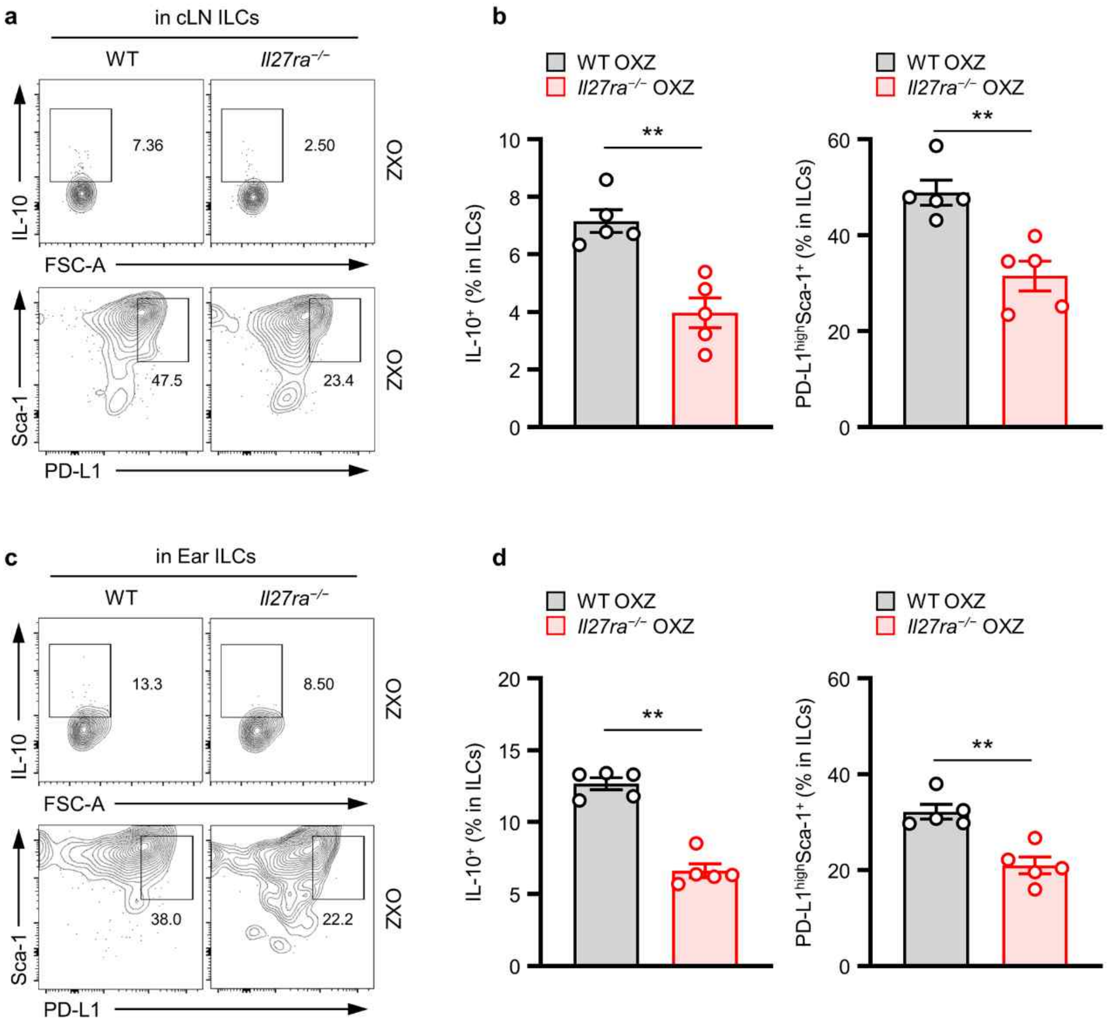

Supplementary Fig 6. The population of IL-10-producing PD-L1^{high}Sca-1^{+} ILCs was decreased in *Il27ra*$^{-/-}$ mice with CHS. a−d Representative plot images show IL-10^{+} ILCs and PD-L1^{high}Sca-1^{+} ILC subsets in cLN (**a**) and ear (**c**) from WT or *Il27ra*$^{-/-}$ CHS mice. The histograms show the frequencies of IL-10^{+} ILCs and PD-L1^{high}Sca-1^{+} ILC subsets in cLN (**b**) and ear (**d**) from WT or *Il27ra*$^{-/-}$ CHS mice (n=5). The results are expressed as representative images (**a**, **c**) and the mean ± SEM (**b**, **d**). **$p < 0.01$ by Student's *t*-test.

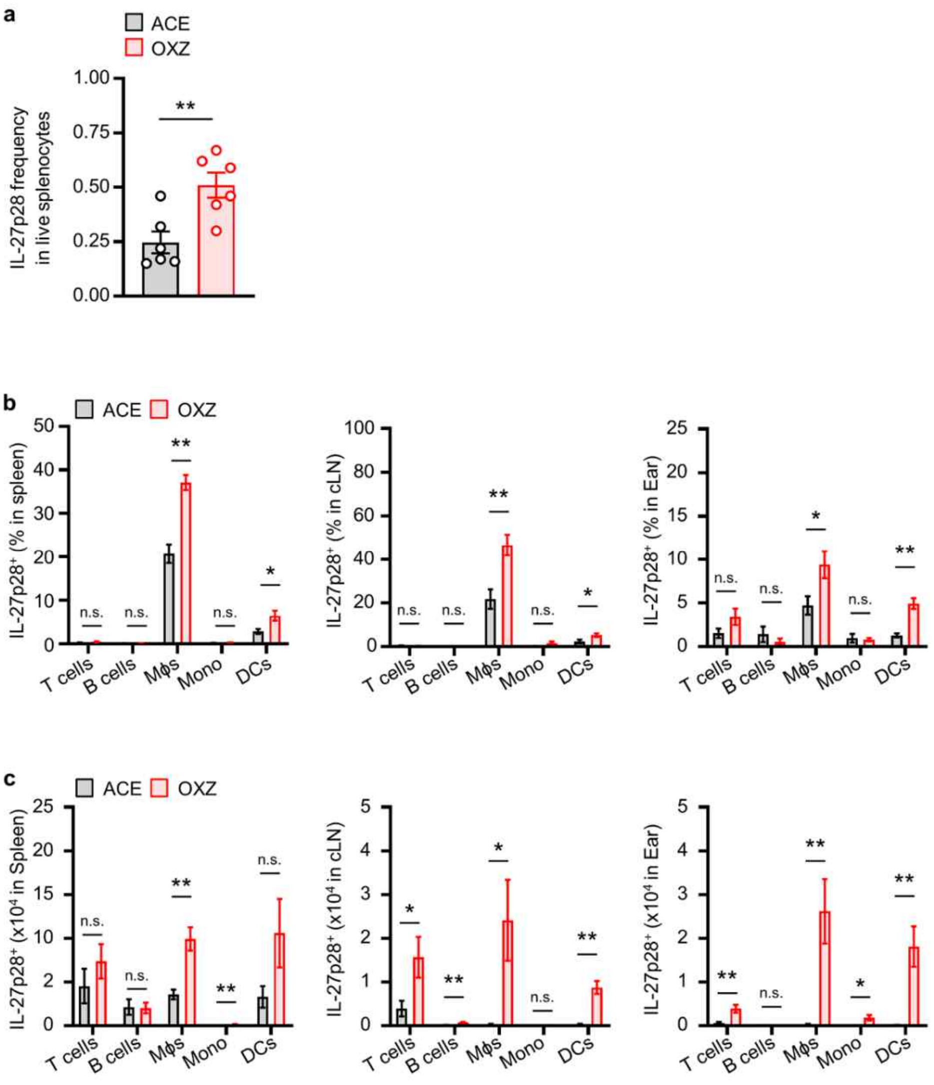

Supplementary Fig 7. Identification of IL-27-producing cells in mice with CHS. a Two days after OXZ challenge, the histograms show the frequency of IL-27p28^{+} cells in live whole splenocytes from ACE or OXZ-induced mice (n=6). **b**, **c** The histograms show indicated IL-27p28^{+} leukocytes (CD3^{+} T cells, CD19^{+} B cells, CD11b^{+}F4/80^{+} macrophages, CD11b^{+}F4/80^{-} monocytes and CD11c^{+} dendritic cells) in spleen, cLN and ear. The histograms show the frequencies (**b**) and numbers (**c**) of IL-27p28^{+} leukocytes in spleen, cLN and ear (n=6). The data are expressed as the mean ± SEM from two independent experiments (n=3 per group for each experiment). *p < 0.05, **p < 0.01, and n.s., not significant versus ACE group by Student's *t*-test.

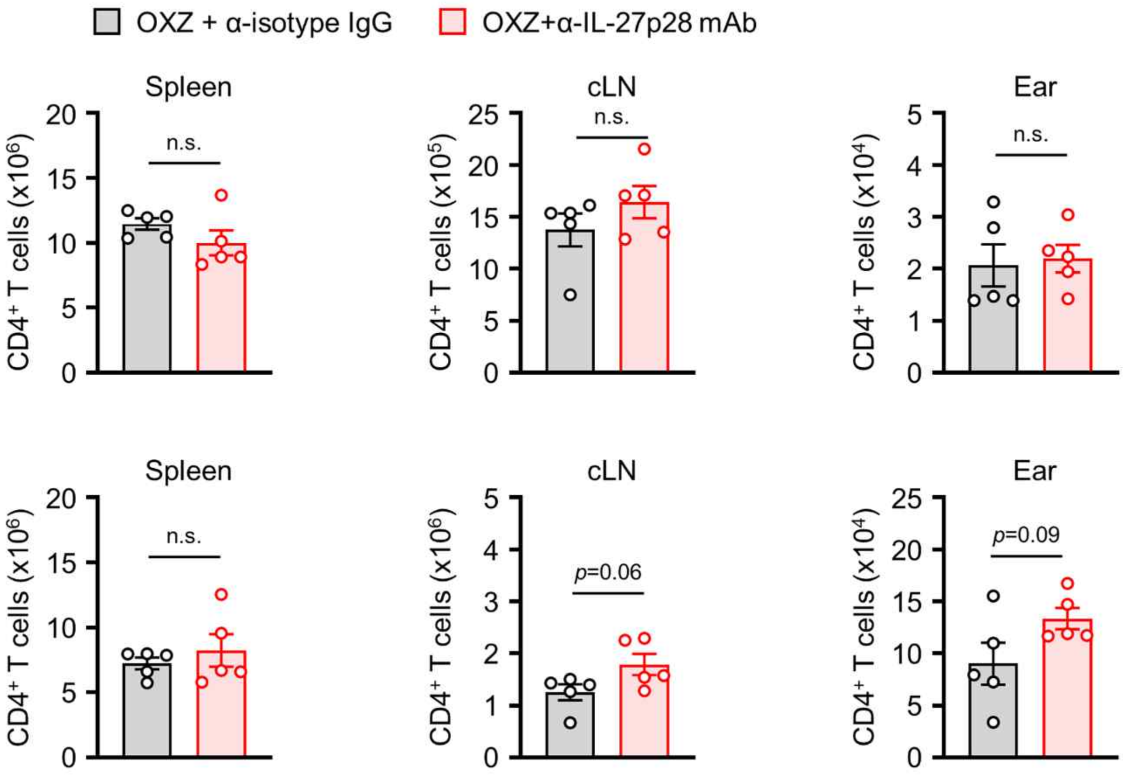

Supplementary Fig 8. Evaluation of changes in T subsets following IL-27p28 mAb neutralization in OXZ-induced CHS mice. On days 4 and 5 after sensitization of CHS mice, either isotype IgG or IL-27p28 mAb (250 μg/mouse) was administered i.p. injection. Two days after OXZ challenge, histograms show the numbers of $CD4^+$ Th cells and $CD8^+$ CTLs in spleen, cLN, and ear from WT CHS mice with or without anti-IL-27p28 mAb treatment (n=5). n.s., not significant versus OXZ + α-isotype IgG group by Student's *t*-test.

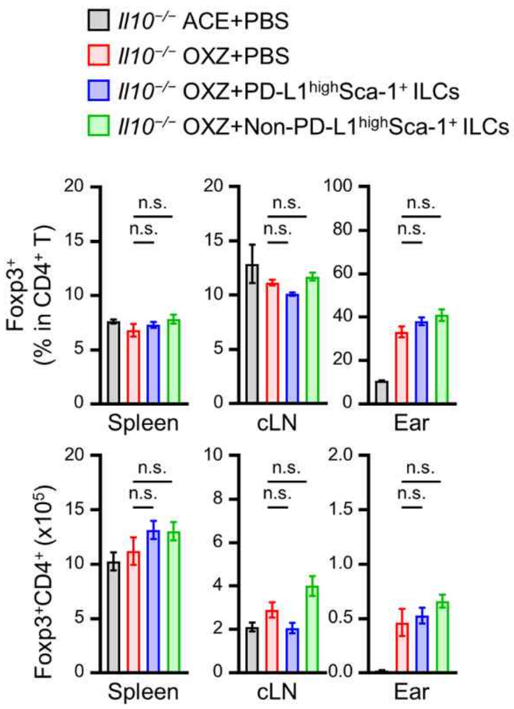

Supplementary Fig 9. The adoptive transfer of PD-L1^{high}Sca-1^{+} ILC subset does not induce the population of Foxp3^{+} Treg Cells. The histograms for the frequencies (upper) and numbers (lower) of Foxp3^{+} regulatory T cells in spleen, cLN, and ear from *Il10*$^{-/-}$ CHS mice transferred with WT PD-L1^{high}Sca1^{+} or PD-L1$^{low/-}$Sca-1^{-} ILC subsets. The data are expressed as the mean ± SEM from two independent experiments (n≥3 per group for each experiment). n.s., not significant versus *Il10*$^{-/-}$ OXZ+PBS group by Student's *t*-test.
